# Vocal display rules in everyday life: when is it appropriate to laugh, sigh or scream?

**DOI:** 10.1098/rstb.2025.0151

**Published:** 2026-09-24

**Authors:** Roza G. Kamiloğlu

**Affiliations:** ^1^ Department of Experimental and Applied Psychology, Vrije Universiteit Amsterdam, Amsterdam, The Netherlands; ^2^ Institute Brain and Behaviour Amsterdam (iBBA), Vrije Universiteit Amsterdam, Amsterdam, The Netherlands

**Keywords:** nonverbal vocalizations, display rules, experience sampling, situation cues, psychological situation characteristics

## Abstract

The appropriateness of common nonverbal vocalizations is guided by social norms, known as display rules. This preregistered, 7-day experience-sampling study examined how perceived appropriateness varies across situations. A total of 142 adults completed three surveys per day (2670 surveys total; 3821 vocalizations), rating appropriateness alongside situational cues and psychological situation characteristics. Laughter and sighs were most often judged appropriate, whereas screams and retches were least. Appropriateness was higher at home and in social places (e.g. parties) than in work/school or public settings (e.g. library), and higher with close ties than with weak ties or strangers. Formality and power were associated with lower appropriateness, whereas sociality and positivity were associated with higher appropriateness. Effects were vocalization-specific: for example, laughter was considered more appropriate in positive, social and brisk contexts, but less appropriate in formal and high-power contexts. By charting when and where vocalizations are judged acceptable, these findings outline situational principles of vocal display rules in everyday life.

This article is part of the theme issue ‘Mechanisms, development, phylogeny and functions of emotional expressions’.

## 1. Introduction

In everyday life, people laugh with friends, cheer when their team wins and cry in moments of loss. We also sigh in relief, moan in pleasure and scream in alarm. Such nonverbal vocalizations are common. For example, laughter occurs about every two minutes in conversation [1]. They also serve important functions: screams alert others to danger [2], cries elicit care from others [3] and sighs help regulate physiological state [4]. Yet how vocalizations are received depends on how well they align with the social norms of a given situation. A belly laugh that feels natural in a café might bring people together, yet in a boardroom, it would likely draw strange looks or even disapproval. Similarly, a groan of frustration shared over a glass of wine might deepen a friendship, but on a first date, it could come across as overly intimate. These patterns reflect display rules: social norms that guide people to amplify, tone down, mask or substitute expressions to fit situational demands [5,6]. This study aims to map when and where vocalizations like laughs, sighs and screams are considered appropriate in everyday life.

### (a) Emotion expression norms across situations

Display rules are cognitive representations of social conventions learned early in life [7] that coordinate emotional expression according to social context [5,8]. Display rules for nonverbal emotional expressions vary across contexts [9,10]: People are generally more expressive in private than in public settings, where norms call for greater control over emotional displays [11,12]. People are also emotionally more expressive with close others than with strangers [13,14]. However, much of this evidence relies on broad categories such as private versus public or close versus distant others, and it does not identify which specific situational features guide display rules.

Psychological research conceptualises situations via two kinds of information [15]. First, situation cues represent the observable, physical elements of the environment: persons and interactions (Who?), objects, events and activities (What?) and spatial locations (Where?). These cues might be associated with different expression norms. For example, restaurants, libraries or home might carry different behavioural expectations. Second, psychological situation characteristics capture how people subjectively experience situations along key dimensions. These characteristics refer to the subjective perception of the situation's meaning: for example, the same physical setting may be experienced as formal or informal depending on the nature of the interaction. These perceived characteristics define the social meaning of a situation and likely shape display rules: a highly formal situation may restrict expression, while a highly social situation may encourage it. The present study examines seven psychological situation characteristics selected to capture features likely to shape vocal display rules. Three dimensions were drawn from the DIAMONDS taxonomy [15], namely positivity, negativity and sociality, which have been validated as core features of psychological situations. Four additional dimensions were selected for their theoretical relevance to vocal expression: formality, which captures communicative registers that prescribe restraint [16]; power, which indexes hierarchical asymmetries linked to expression regulation and sanction risk [17]; briskness, which reflects situational energy and activation that may license louder or more exuberant vocalizations [18]; and novelty, which captures unexpectedness that may elicit spontaneous vocal reactions such as gasps.

### (b) Vocal display rules

Vocalizations offer a unique and understudied domain for studying display rules. They form a heterogeneous repertoire that conveys diverse emotions [19,20] and serves different social and biological functions [21], yet can be readily identified in everyday situations without expert training. If display rules simply prescribe general emotional restraint, all vocalizations should be governed by similar norms. However, if display rules are sensitive to the function of specific expressions, vocalizations serving affiliative functions (e.g. laughter [22]) may be governed by different norms than those serving alarm (e.g. screams) or physiological regulation (e.g. sighs [4]). Examining these patterns can inform whether display rules represent general prescriptions for restraint or more nuanced expectations calibrated to what different expressions signal. Beyond mapping, understanding which situational features shape appropriateness judgments can inform theories of social functioning [18] and identify targets for future experimental research on how situations causally influence display rules.

One study assessed the perceived appropriateness of 10 nonverbal vocalizations across four countries [23]: laughter and sighs were rated as most appropriate, whereas groans, moans and growls were less appropriate. Appropriateness was generally higher in private settings and with close others, though effects varied by vocalization type. This initial work has two limitations. First, it examined only broad context categories, missing the rich array of situation cues and psychological characteristics that may influence appropriateness. Second, the research relied on hypothetical judgments about imagined scenarios, which can test whether specific contexts affect appropriateness but cannot reveal which vocalizations naturally occur in which contexts or how appropriateness varies across the full ecology of everyday situations [23]. The present study addresses these limitations using experience sampling, reducing recall bias and capturing variability across real-world contexts.

### (c) The present study

The present study examined how vocal display rules vary across everyday situations using a 7-day experience-sampling design. Three times per day, participants reported the current situational features, which vocalizations occurred, and how appropriate those vocalizations are generally considered. Two kinds of situational information were modelled: (a) situation cues, including location and interaction partner; and (b) psychological characteristics, namely positivity, negativity, sociality, formality, power, briskness and novelty [15]. Because vocal display rules have not previously been examined across everyday situations, it remains unclear which situational features are associated with appropriateness for different types of vocalizations. The present study aims to map this landscape; accordingly, the hypotheses predict systematic variation across situational factors.

**Hypothesis 1 (Vocalization type).** Perceived appropriateness will vary across vocalization types.

**Hypotheses H2a, H2b and H2c (Situational main effects).** Appropriateness will vary across locations (home, work/school, social places and public places; H2a); interaction partners (close ties, weak ties and strangers) and when alone (H2b); and with psychological characteristics, including positivity, negativity, sociality, formality, power, briskness and novelty (H2c).

**Hypotheses H3a, H3b and H3c (Vocalization–situation interactions).** The associations between appropriateness and (a) location (H3a), (b) interaction partner (H3b) and (c) psychological characteristics (H3c) will differ across vocalization types.

## 2. Methods

### (a) Participants

All participants provided informed consent. Eligibility criteria required that participants be (i) aged 18 years or older, (ii) have regular access to an internet-enabled smartphone and (iii) report no hearing impairment.

A target sample of 150 adults was preregistered based on both statistical power requirements and feasibility constraints. With each participant providing up to 21 observations (three surveys × 7 days) and accounting for an estimated 20–30% noncompliance, simulation studies for multilevel models indicate that this sample size provides over 80% power to detect medium-sized effects (*d* = 0.3–0.5) at both within-person and between-person levels [24]. This also aligns with experience sampling method (ESM) methodology recommendations that 100+ participants with 15+ observations each provide adequate power [25].

Participants were recruited through the (masked for review) subject pools (*n* = 109; 72.7%) and convenience sampling via the researcher's extended networks, mainly including current and former students as well as students and lab members of colleagues (*n* = 41; 27.3%), between April and September 2025. Of the 150 participants recruited, four dropped out after the intake session, and four were excluded for completing <30% of the surveys; as such, low compliance does not provide sufficient within-person observations to contribute meaningfully to multilevel models. Sensitivity analyses including these excluded participants yielded virtually identical results (supplementary material, S1).

The final sample consisted of 142 participants (107 women, 33 men, one nonbinary, one prefer not to say; *M*_age_ = 23.29, *SD*_age_ = 3.98, range = 18–53). Participants represented 39 nationalities (30% Dutch, 70% other; see table S1, supplementary material for full breakdown). They completed a median of 20 surveys (IQR = 18–21) with an average compliance rate of 99.1%. All survey items required a response before submission, eliminating item-level missing data within completed surveys. After uncompleted surveys were removed (*n* = 21), the total dataset included 2670 effective surveys containing 3821 vocalization observations. Participants received €10 per hour compensation paid pro rata, plus a completion bonus for responding to 17 or more of 21 surveys.

### (b) Materials

#### (i) Intake survey

The intake survey collected demographic information, including age, gender and nationality, mobile phone numbers for survey prompting, confirmations of eligibility criteria (see Participants), and included a practice trial of the experience-sampling survey to ensure comprehension.

#### (ii) Experience-sampling survey

Each survey took approximately 5 minutes and comprised 10 to 20 items, depending on the number of vocalizations observed (see supplementary material, S2, for complete survey). First, participants reported their current location and interaction partners. Second, they rated seven psychological characteristics of their current situation. Third, they indicated which vocalizations (if any) they had observed in that context. Finally, for each observed vocalization, they rated its perceived appropriateness in that specific context.

##### Location

Location was assessed with a single-choice item, *Where are you right now?* (12 options plus free text) adapted from previous ESM work [26,27]. Following Chung *et al.* [26], responses were recoded into four categories based on primary function: Home (private space; *n* = 1405), Work/School (institutional settings; *n* = 386), Social Place (venues for socializing with known others, e.g. bar, party, friend's house; *n* = 244) and Public Place (venues for individual activities in shared spaces, e.g. café, gym, library; *n* = 570). Free-text entries were reviewed and recoded where possible; uncodeable entries were excluded (see supplementary material, S3).

##### Interaction partners

Interaction partners were assessed with a multiple-select item, *Who are you with right now?* (eight options plus free text) adapted from prior ESM studies [26,27]. Responses were recoded into four categories based on relationship closeness [26]: Alone (*n* = 933), Close Ties (e.g. family, friends and significant other; *n* = 1288), Weak Ties (e.g. classmates and co-workers; *n* = 318) and Strangers (*n* = 371). Each selection was treated as a separate observation; 10.3% of surveys involved multiple partner categories. In these cases, the single appropriateness rating for that vocalization appears in multiple partner categories. A sensitivity analysis excluding these multi-partner observations yielded robust results (supplementary material, S4). Free-text entries were reviewed and recoded where possible, and the remaining Other were excluded from analysis (see supplementary material, S5).

##### Psychological situation characteristics

Participants rated seven dimensions on 5-point scales (1 = *Not at all*, 5 = *Extremely*) to match validated instruments and minimise respondent burden across repeated assessments. Three dimensions were adapted from validated situation-taxonomy measures based on the DIAMONDS framework [15,28] and its ultra-brief S8 items [26]: Positivity (*This context is positive*), Negativity (*This context is negative*) and Sociality (*Social interactions are possible or required*). Four additional dimensions were: Formality (*This context is formal*), Power (*There is a clear power difference between people in this context*), Briskness (*This context is energetic or lively*) and Novelty (*This context is unusual or unexpected*).

##### Vocalizations

Participants indicated which nonverbal vocalizations they had observed in the current context, including both their own and others’ vocalizations. This inclusive approach was adopted to capture vocalizations across all contexts, including when participants were alone. Response options included ten vocalization types (Cry, Gasp, Groan, Grunt, Laugh, Moan, Retch, Growl, Scream and Sigh) derived from established taxonomies [21,29], plus Other (free text) and None observed. Multiple selections were permitted. Free-text entries (*n* = 229) and surveys with no vocalizations observed (*n* = 625) were excluded from the main analyses (see supplementary material, S6, for full details).

##### Perceived appropriateness

For each selected vocalization, participants rated perceived appropriateness using a single item adapted from the Vocalization Display Rules Assessment Protocol (VODRAP; [23]): *In general, how appropriate do you think most people would find expressing (vocalization) in this context?* Ratings were provided on a 9-point scale (1 = Very inappropriate and 9 = Very appropriate), consistent with the VODRAP protocol; this wider scale was chosen to capture fine-grained variation in the primary dependent variable. This operationalises appropriateness as perceived social norms (intersubjective display rules) rather than personal appropriateness judgments, consistent with foundational approaches to display-rule assessment [8,13].

### (c) Experimental procedure

The study comprised two phases: an online intake session and a 7-day experience-sampling period. Participants first attended a Zoom (zoom.us) meeting, during which the procedure was explained, including the instruction that, upon receiving each prompt, participants should consider vocalizations observed within approximately the last 30 minutes. Questions were addressed, and informed consent was obtained. Immediately afterward, they completed the intake survey in Qualtrics (qualtrics.com), which included demographics, eligibility checks and a practice trial (see §2b). Participants could still ask questions after the practice trial. The experience-sampling phase began the following day.

During the 7-day sampling period, participants received three survey prompts per day, scheduled on a stratified random basis within a 14-hour waking window (09:00–23:00). Each day was divided into morning (09:00–13:00), afternoon (13:00–18:00) and evening (18:00–23:00) blocks, with one survey randomly scheduled per block. SMS notifications were delivered via Spryng (spryng.nl), each containing a unique Qualtrics survey link. Participants were instructed to complete surveys within 1 hour of notification; while later responses were not excluded, the importance of timely completion was emphasised to maximise situational variability.

Each survey followed a fixed structure (supplementary material, figure S1). Participants first completed situation cue questions (location and interaction partners) and rated psychological situation characteristics. They then indicated which vocalizations, if any, they had observed in that situation. For each selected vocalization, participants provided appropriateness ratings.

### (d) Statistical analysis

#### (i) Preliminary analyses and data structure

Preliminary analyses included descriptive statistics and intraclass correlation coefficients (ICCs) for variance partitioning. For the main analysis, multilevel models were used to account for the nested data, with vocalization-specific appropriateness rating (Level 1) nested within participants (Level 2). Multiple ratings from the same prompt were modelled as independent Level-1 observations, clustered within participants.

#### (ii) Variable coding

Appropriateness was treated as continuous (1–9). Categorical predictors were effect-coded. Psychological situation characteristics were person-mean centred to estimate within-person effects, with person means (grand-mean centred) included to model between-person differences.

#### (iii) Analytical strategy

H1 tested vocalization type effects with Tukey-adjusted pairwise contrasts. H2 used separate models for (a) location, (b) interaction partners and (c) seven psychological dimensions entered simultaneously. H3 tested interactions between vocalization type and situational factors; analyses were restricted to the seven vocalizations with sufficient observations for interaction analysis (Laugh, Sigh, Gasp, Groan, Scream, Grunt and Moan); Cry, Growl and Retch were excluded due to low overall frequencies (all fewer than 150 observations). Interactions with psychological dimensions were tested separately for each dimension. Significant interactions were probed using simple slopes and estimated marginal means. Multiple comparisons were corrected using Holm-Bonferroni within each hypothesis family and across the seven psychological dimensions for H3c. Regarding variance levels: the ICC captures stable between-person differences, H1 reflects variation across expression types, and all situational effects (H2 and H3) capture within-person variation. For H2c, person-mean centred scores estimate within-person effects and grand-mean centred person means capture between-person differences (see Variable Coding).

Linear mixed-effects models were fit using lme4 [30] with random intercepts for participants. Model comparisons used likelihood-ratio tests (nested models) and AIC/BIC (Akaike/Bayesian Information Criterion; non-nested). Fixed-effect inference used Satterthwaite's approximation [31]. Effect sizes include Cohen's *f* (omnibus), Cohen's *d* (pairwise), and *η*² (interactions). Variance explained was reported as *R*² marginal [32]. Statistical significance was set at *α* = 0.05.

## 3. Results

### (a) Descriptive results and building multilevel models

Null models revealed that 21.1% of variance was attributable to stable between-person differences (ICC = 0.211, 95% CI [0.182, 0.239]), and 78.9% to within-person variation across prompts. This large within-person component is expected for context-dependent ratings and justifies multilevel modelling. Between-person ICCs for psychological dimensions ranged from 0.195 to 0.233, indicating meaningful individual differences in how participants experienced and rated situational features. Descriptive statistics for all study variables are reported in supplementary material, table S2, and model fit indices for all hypothesis models, including AIC, BIC, R²marginal, R²conditional, and likelihood ratio tests comparing each model to the null baseline, are reported in table S3.

Table 1 presents the percentage of surveys in which each vocalization was observed, broken down by location and interaction partner. Vocalization production varied substantially across contexts. Laughter was observed in 75.4% of surveys in social places but 30.2% at home, and in 67.9% of surveys with close ties but 13.1% when alone. Sighs were most frequently observed at work/school (46.6%) and with weak ties (48.4%). Screams were most frequent in social places (23.8%) and with strangers (17.8%) or close ties (15.5%). All vocalizations were observed more frequently with others present than when alone.

**Table 1.** Percentage of surveys in which each vocalization was observed, by location (panel A) and interaction partner (panel B). Values represent the percentage of surveys within each context category in which the vocalization was reported. Vocalizations are ordered from most to least frequently observed across the full dataset. Surveys in which no vocalization was observed (23.4%) are included in the denominators.

panel A: location				
vocalization	home (*n* = 1405)	work/school (*n* = 386)	social place (*n* = 244)	public place (*n* = 570)
laugh	30.2	57.8	75.4	63.3
sigh	30.5	46.6	34.8	37.4
gasp	9.9	13.2	21.3	19.1
groan	9.7	11.9	13.1	15.1
scream	7.1	9.3	23.8	17.9
grunt	5.8	9.3	8.6	15.1
moan	7.3	4.9	8.2	8.9
cry	3.6	3.9	6.6	9.6
growl	2.0	2.3	1.6	4.0
retch	1.0	3.4	1.6	2.8
**panel B: interaction partner**				
**vocalization**	**alone (*n* = 933)**	**close ties (*n* = 1288)**	**weak ties (*n* = 318)**	**strangers (*n* = 371)**
laugh	13.1	67.9	61.3	59.8
sigh	22.7	41.3	48.4	40.7
gasp	6.6	18.9	14.8	21.8
groan	6.3	15.6	11.0	17.8
scream	4.3	15.5	11.0	17.8
grunt	4.5	10.7	10.4	16.4
moan	3.8	10.2	6.9	10.5
cry	3.3	6.4	5.3	10.8
growl	0.8	2.6	3.1	4.9
retch	0.5	2.6	3.1	2.2

### (b) H1: Vocalization type

**Figure 1.**
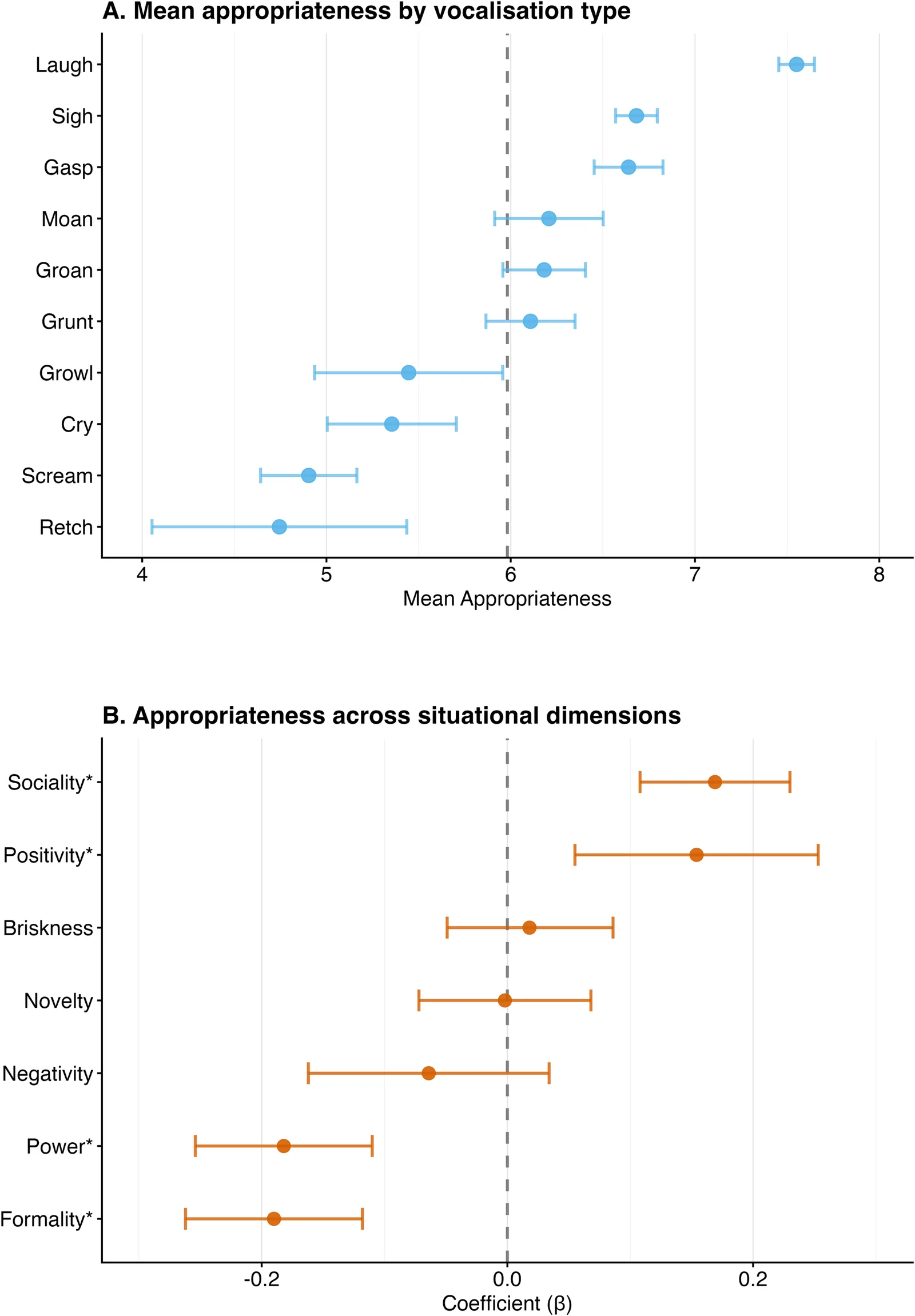
(A) Mean appropriateness ratings for each vocalization type, ordered from most to least appropriate. Error bars represent standard deviations. The dashed vertical line represents the overall mean appropriateness across all vocalizations. (B) Standardised regression coefficients (β) showing associations between psychological situation dimensions and vocalization appropriateness. Error bars represent 95% confidence intervals. *p < 0.05.

Vocalization type accounted for 16.1% of the variance in appropriateness, *F*(9, 3721.1) = 101.19, *p* < 0.001, Cohen's *f* = 0.495. Laughter emerged as the most appropriate vocalization, followed by a sigh and a gasp. Mid-range vocalizations included moan, groan and grunt, and lower-rated vocalizations were growl, cry, scream and retch. Appropriateness means, and standard deviations per vocalization are shown in figure 1A; complete pairwise comparisons are provided in table S4, supplementary material.

### (c) H2a: Location

Location accounted for 2.7% of the variance in appropriateness across all vocalization types, *F*(3, 3694.5) = 38.83, *p* < 0.001, Cohen's *f* = 0.18. Home (*M* = 6.94, s.d. = 1.97) and social places (*M* = 6.92, s.d. = 2.05) were rated most appropriate and did not differ significantly (*d* = −0.07, *p* = 0.590). Public places (*M* = 6.27, s.d. = 2.11) and work/school settings (*M* = 6.37, s.d. = 2.01) were rated least appropriate and also did not differ (*d* = 0.00, *p* = 1.000). Pairwise contrasts showed that both home and social places were rated more appropriate than public places and work/school settings (all *p*s < 0.001, *d* = 0.36–0.43). Distributions are shown as a boxplot in figure S2, supplementary material; complete pairwise contrasts are provided in table S5, supplementary material. A within–between decomposition confirmed that these effects reflect within-person situational variation rather than between-person differences in context exposure (see supplementary material, S7).

### (d) H2b: Interaction partner

Interaction partner accounted for 1.7% of the variance in appropriateness across all vocalization types, *F*(3, 4385.9) = 25.98, *p* < 0.001, Cohen's *f* = 0.133. Close ties were rated as most appropriate (*M* = 6.76, s.d. = 2.07), followed by being alone (*M* = 6.51, s.d. = 2.03). Strangers (*M* = 6.30, s.d. = 1.99) and weak ties (*M* = 6.46, s.d. = 2.01) were rated as least appropriate and did not differ significantly (*d* = 0.05, *p* = 0.846). Pairwise contrasts showed that close ties were rated as more appropriate than both strangers (*d* = 0.36, *p* < 0.001) and weak ties (*d* = 0.31, *p* < 0.001). Being alone also differed significantly from being with strangers (*d* = 0.20, *p* = 0.006). Distributions are shown as a boxplot in figure S3, supplementary material; complete pairwise comparisons are provided in table S6, supplementary material. Within–between decomposition results were consistent (supplementary material, S7).

### (e) H2c: Psychological situation dimensions

Psychological dimensions collectively explained 7.2% of the variance in appropriateness across all vocalization types. Only A likelihood ratio test comparing the full model (including all seven dimensions) to a model with random effects was significant, $\chi^{2}(14)$ = 233.88, *p* < 0.001. In the simultaneous model with person-mean centred psychological dimensions, higher Power (*β* = −0.182, *SE* = 0.037, 95% CI [−0.254, −0.110], *p* < 0.001) and Formality (*β* = −0.190, *SE* = 0.037, 95% CI [−0.262, −0.118], *p* < 0.001) were associated with lower perceived appropriateness, whereas Sociality (*β* = 0.169, *SE* = 0.031, 95% CI [0.108, 0.230], *p* < 0.001) and Positivity (*β* = 0.154, *SE* = 0.051, 95% CI [0.055, 0.253], *p* = 0.002) were associated with higher appropriateness. Negativity (*β* = −0.064, *SE* = 0.050, 95% CI [−0.162, 0.034], *p* = 0.203), Briskness (*β* = 0.018, *SE* = 0.035, 95% CI [−0.049, 0.086], *p* = 0.596) and Novelty (*β* = −0.002, *SE* = 0.036, 95% CI [−0.072, 0.068], *p* = 0.955) were not significant. Standardised coefficients are shown in figure 1B, and within-person variability across dimensions is illustrated in figure S4, supplementary material.

### (f) H3a: Vocalization × location

**Figure 2.**
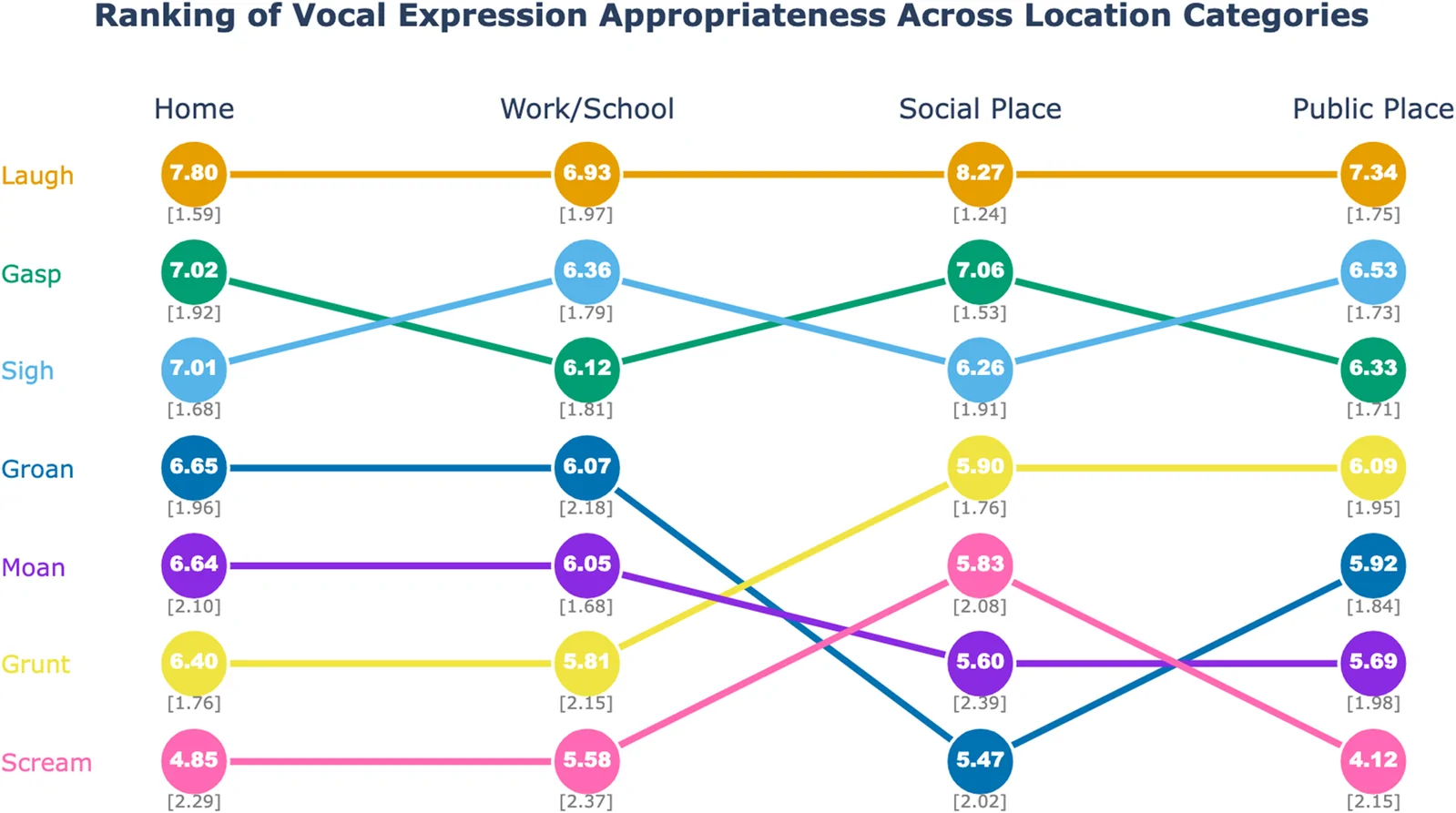
Ranking of vocalization appropriateness across location categories. Numbers in circles show mean appropriateness ratings; numbers in brackets show standard deviations. Connecting lines illustrate ranking shifts between home, work/school, social places and public places.

The vocalization type × location interaction accounted for 3.0% of the variance in appropriateness ratings, *F*(18, 3327.37) = 5.66, *p* < 0.001, *η*² = 0.030. Laugh demonstrated the strongest location sensitivity, with all pairwise contrasts significant (all *p*s < 0.001). Social places were rated significantly higher than all other locations (versus home: *t* = −3.95; versus public places: *t* = −7.03; versus work/school: *t* = 10.04), and home was rated higher than both public places (*t* = 3.90) and work/school (*t* = 7.78). Scream showed significant context effects, with the largest difference between social places and public places (*t* = −6.94, *p* < 0.001). Sigh exhibited moderate location effects, with home consistently rated higher than all other locations (versus public places: *t* = 3.23, *p* = 0.006; versus social places: *t* = 3.03, *p* = 0.010; versus work/school: *t* = 4.32, *p* < 0.001). Gasp showed significant differences between social places and both public places (*t* = −3.12, *p* = 0.009) and work/school (*t* = 3.46, *p* = 0.003). Groan was rated higher at home than in public places (*t* = 3.43, *p* = 0.003) and social places (*t* = 3.75, *p* = 0.001), and moan was rated higher at home than in public places (*t* = 4.20, *p* < 0.001) and work/school (*t* = 2.64, *p* = 0.041). Grunt showed no significant contrasts across locations. Figure 2 shows the ranking of appropriateness per location, and complete results are provided in table S7, supplementary material.

### (g) H3b: Vocalization × interaction partner

**Figure 3.**
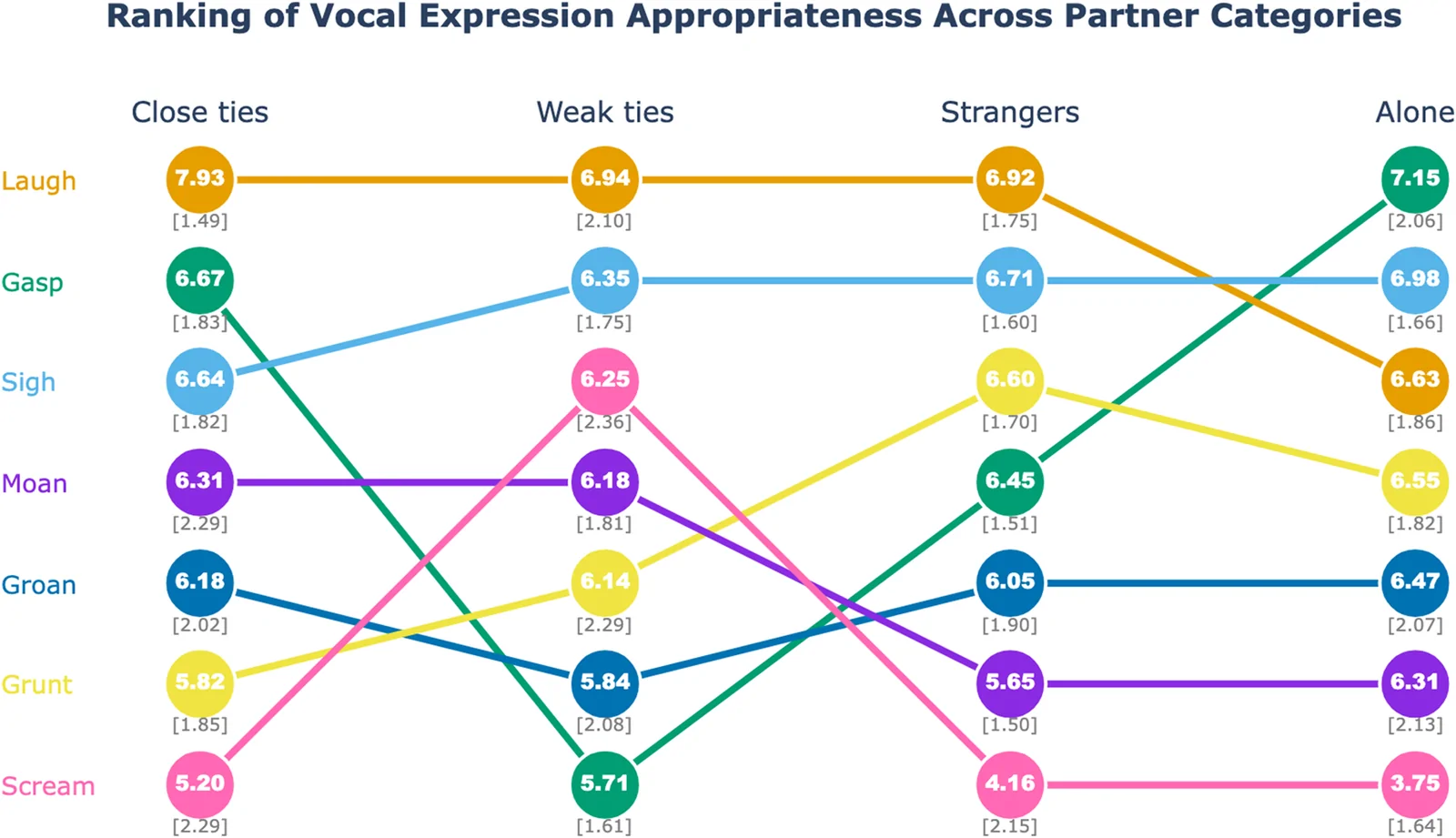
Ranking of vocalization appropriateness across interaction partner categories. Numbers in circles show mean appropriateness ratings; numbers in brackets show standard deviations. Connecting lines illustrate ranking shifts between close ties, weak ties, strangers and being alone.

The interaction between vocalization type and interaction partner accounted for 3.4% of the variance in appropriateness ratings, *F*(18, 3974.6) = 7.74, *p* < 0.001, *η*² = 0.034. Laugh demonstrated the strongest partner effects, with close ties rated significantly higher than all other contexts (versus alone: *t* = −7.59, *p* < 0.001; versus strangers: *t* = 7.45, *p* < 0.001; versus weak ties: *t* = 9.67, *p* < 0.001). The difference between close ties and weak ties represented the largest partner effect across all vocalizations. Scream showed significant partner sensitivity, with close ties rated higher than both alone (*t* = −3.98, *p* < 0.001) and strangers (*t* = 4.38, *p* < 0.001). Sigh exhibited a distinct pattern, with being alone rated higher than all other partner types (versus close ties: *t* = 3.21, *p* = 0.005; versus strangers: *t* = 3.69, *p* = 0.001; versus weak ties: *t* = 3.96, *p* < 0.001). Gasp, groan, grunt and moan showed no significant partner effects. Figure 3 shows vocalization appropriateness rankings across interaction partner types; complete results are provided in table S8, supplementary material.

### (h) H3c: Vocalization × psychological situation dimensions

Simple-slope tests (Holm–Bonferroni adjusted) indicated that associations between psychological dimensions and appropriateness varied by vocalization (supplementary material, table S9). For positivity, appropriateness was higher for laugh (*β* = 0.255, *SE* = 0.075, 95% CI [0.052, 0.458], *p* = 0.005) and for scream (*β* = 0.388, *SE* = 0.138, 95% CI [0.018, 0.759], *p* = 0.029). For sociality, appropriateness was higher for laugh (*β* = 0.394, *SE* = 0.051, 95% CI [0.256, 0.531], *p* < 0.001) and scream (*β* = 0.296, *SE* = 0.099, 95% CI [0.030, 0.562], *p* = 0.014), but lower for grunt (*β* = −0.355, *SE* = 0.105, 95% CI [−0.636, −0.073], *p* = 0.004). Formality was associated with lower appropriateness for laugh (*β* = −0.320, *SE* = 0.057, 95% CI [−0.473, −0.167], *p* < 0.001) and moan (*β* = −0.401, *SE* = 0.151, 95% CI [−0.806, 0.004], *p* = 0.046). For power, only laugh showed a decrease (*β* = −0.197, *SE* = 0.057, 95% CI [−0.350, −0.045], *p* = 0.004). Briskness related to higher appropriateness for laugh (*β* = 0.172, *SE* = 0.057, 95% CI [0.019, 0.325], *p* = 0.015) and scream (*β* = 0.392, *SE* = 0.107, 95% CI [0.103, 0.681], *p* = 0.002). Figure S5 in supplementary material shows a heatmap of simple-slope coefficients (*β*) for each characteristic × vocalization.

A summary of all hypothesis tests is provided in table S10, supplementary material. Furthermore, controlling for nationality did not alter the pattern of results, though Dutch participants rated appropriateness higher overall (supplementary material, table S11).

## 4. Discussion

This preregistered experience-sampling study demonstrated that laughter and sighs consistently emerged as most appropriate, while screams and retches were perceived as least appropriate in everyday life. Appropriateness varied systematically across locations, interaction partners and psychological situation characteristics, with effects differing across vocalization types.

### (a) Appropriateness patterns across vocalizations

Nonverbal vocalizations serve distinct intra- and interpersonal functions [21], which may explain why their appropriateness varies across social contexts. Laughter emerged as the most appropriate vocalization, which may reflect its role as a play signal that facilitates social bonding [22,33] and communicates companionship and nonthreatening intent [34]. As a signal broadly interpreted as friendly and affiliative, laughter may be widely acceptable across diverse social contexts. Sighs and gasps followed. Sighs may serve multiple functions [4], including physiological regulation (reinflating alveoli) and emotional regulation (providing a psychological reset from stress), while potentially conveying emotional states such as relief or boredom to others [35,36]. Gasps involve a sharp inhalation signalling surprise or shock. Their consistent acceptability suggests these vocalizations are primarily physiological or self-regulatory and therefore less socially demanding.

Screams and retches received the lowest appropriateness ratings. Both vocalizations disrupt social interaction, likely explaining their low appropriateness. Screams function as high-intensity alarm signals [2,21] that occupy a unique acoustic niche characterised by roughness, rapidly activating fear circuits in listeners [2] and disrupting ongoing interaction. Retches signal contamination or illness, functioning as part of the disease-avoidance system to minimise pathogen exposure [37], triggering disgust and avoidance responses in others [38] and thereby disrupting interaction.

### (b) Situational cues: location and interaction partner

Perceived appropriateness varied systematically across locations and interaction partners, extending classic display rule principles [5,8,13] to specific situational categories. Home and social places (e.g. friends’ houses and bars) were rated most appropriate, whereas work/school and public settings (e.g. libraries, stores) were least appropriate. These differences may reflect that home and social places offer privacy and shared social goals, while work/school and public settings involve focus on activities and uninvolved bystanders. Similarly, close ties were rated most appropriate, being alone fell in between, and weak ties/strangers were least appropriate, possibly because close relationships provide shared understanding, whereas distant others raise the chance of misinterpretation.

These situational effects varied across vocalization types. Laughter was rated highest in social places (versus all other locations) and with close ties (versus all other partner types). This aligns with its role in facilitating social connection [22,33,34], suggesting that laughter's appropriateness depends particularly on situations that support interpersonal bonding. Screams’ appropriateness was rated highest in social places and with close ties. This may reflect that screams not only express negative states like fear, but also positive high-arousal emotions like excitement [21,39]. In contrast, sighs showed a distinct pattern: they were rated higher when alone compared to all partner types (close ties, strangers and weak ties). This reflects that sighs serve intrapersonal regulatory functions [4,35,36] rather than social communication, making them most appropriate in solitary contexts. Moans showed higher ratings at home than in public settings, which may reflect their association with more intimate or private contexts.

Production patterns partially aligned with appropriateness: laughter was most frequently observed and rated most appropriate in both social places and with close ties. But sighs were most frequently observed at work/school and with weak ties, contexts where they were rated less appropriate, suggesting that situational triggers (e.g. stress) may elicit sighs regardless of display rules. These divergences highlight that production reflects both normative pressures and situational elicitation.

### (c) Psychological situational dimensions

Formality and power distance in a situation were associated with lower appropriateness, whereas sociality and positivity were associated with higher appropriateness of vocalizations. This pattern is consistent with work showing that formal contexts call for greater emotional control [12,16] and that social settings afford greater expressiveness [12,13]. Hierarchical contexts may similarly impose norms for behavioural restraint [17].

These psychological dimension effects varied across vocalizations. Laughter was rated more appropriate in positive, social and energetic situations, but less appropriate in formal and high-power situations. This is consistent with laughter's role as a socially regulated bonding signal [20] that may face stricter norms in hierarchical settings. Screams were rated more appropriate in positive, social and brisk contexts. This aligns with evidence that screams serve not only alarm functions but also celebratory and high-arousal positive functions [21,39], making them more acceptable in energetic social situations. Grunts showed the opposite pattern, rated less appropriate as sociality increased. This may reflect that grunts signal exertion or low-arousal states [21] that are less relevant to social interaction. Moans were specifically rated lower in formal contexts, consistent with their association with personal or intimate experiences.

The situational main effects (H2a–H2c) each explained 2–7% of the variance in appropriateness. With an ICC of 0.211, nearly 79% of the variance reflects within-person fluctuation, from which even modest R² values represent systematic effects. Vocalization type explained 16.1%, indicating that what is expressed carries stronger normative weight than where or with whom. Moreover, the vocalization × situation interactions (3.0–3.4%) show that situational effects are vocalization-specific rather than uniformly small: laughter was highly context-sensitive, whereas grunts showed minimal variation across locations.

### (d) Limitations and future directions

First, the sample consisted predominantly of young, female university students. The measure assessed perceived societal norms rather than personal judgments; however, women may tend to endorse different display rules than men [13], and older adults might perceive norms differently than younger adults. Moreover, the situations participants encountered reflect university life, which may involve less formal hierarchies than professional or other adult settings. Additionally, 27.3% of participants were recruited through convenience sampling via the researcher's academic networks and were thus potentially known to the researcher, which may have influenced response patterns (e.g. through heightened compliance or social desirability). Second, the study measured perceived norms rather than actual vocalization. Whether people follow the display rules they report remains an open question. Also, participants reported both their own and others’ vocalizations without distinction. Self-report may favour salient expressions over subtle ones, and reporting may differ for self versus others due to self-presentation concerns. Third, the observational design cannot establish causality. Experimental manipulations would clarify whether situational features directly cause shifts in appropriateness judgments. For example, participants could rate identical vocalizations in experimentally manipulated formal versus informal settings, isolating the causal effect of situations on perceived appropriateness.

An important direction for future research is examining cultural variation. Dutch participants rated appropriateness higher than non-Dutch participants, possibly reflecting greater familiarity with local norms. Previous research [23] found that the magnitude of situational effects varies across four countries. Future research could examine how specific cultural dimensions like tightness–looseness [40] moderate the effects of situational features on appropriateness. For example, cultures with stronger hierarchical norms might show larger effects of power and formality on appropriateness. Furthermore, future research could test the cognitive mechanisms underlying appropriateness judgments, such as inferences about expresser intentions or anticipated social consequences. At the individual level, aspects of cultural background (e.g. acculturation), personality traits, mood, or mental health conditions may also moderate appropriateness judgments across situations.

## 5. Conclusion

This study maps vocal display rules by documenting the perceived appropriateness of vocalizations across everyday situations. Laughter, sighs and gasps were rated most appropriate, while screams and retches were least appropriate. Appropriateness varied with both situational cues (location and relationship closeness) and psychological characteristics (formality, power, sociality and positivity), with patterns differing across vocalization types. These findings suggest that vocal display rules operate through both observable environmental features and subjective psychological meanings, with norms varying by expression type.

## Supplementary Material



## Data Availability

Hypotheses, methods and analyses were preregistered, and all data and analysis scripts are available on the Open Science Framework at [41]. Supplementary material is available online [42].

## Theme

One contribution of 19 to a theme issue ‘Mechanisms, development, phylogeny and functions of emotional expressions’.
