# Supplementary material for: Vocal display rules in everyday life: when is it appropriate to laugh, sigh or scream?
Source: Philos Trans R Soc Lond B Biol Sci. 2026 Sep 24;381(1959):20250151. doi: 10.1098/rstb.2025.0151 (PMC13601882; doi:10.1098/rstb.2025.0151)
Supplement: Supplementary file 1 [file rstb.2025.0151.zip › rstb.2025.0151/rstb20250151_si_001.pdf]

Supplementary Materials

For

Vocal Display Rules in Everyday Life: When Is It Appropriate to Laugh, Sigh, or Scream?

Roza G. Kamiloğlu

Table of Contents

Item	Description	Page
Text S1	Sensitivity analysis for participant exclusion criterion	3
Text S2	Experience sampling survey	5
Text S3	Location assessment	8
Text S4	Sensitivity analysis: excluding multiple interaction partner observations	13
Text S5	Interaction partner assessment	15
Text S6	Vocalisation assessment	17
Text S7	Within-between decomposition of categorical predictors	19
Table S1	Distribution of participant nationalities	20
Table S2	Descriptive results of the main variables	21
Table S3	Model fit indices and comparisons across hypothesis models	22
Table S4	Pairwise comparisons of appropriateness ratings across vocalisation types	24
Table S5	Pairwise comparisons of appropriateness across locations	26
Table S6	Pairwise comparisons of appropriateness across interaction partners	26
Table S7	Vocalisation × Location interactions	27
Table S8	Vocalisation × Interaction partner interactions	29
Table S9	Simple slopes of psychological situation dimensions by vocalisation	31
Table S10	Summary of hypothesis tests	33
Table S11	Effect of nationality (Dutch vs. non-Dutch) as a between-person covariate	34
Figure S1	Experience-sampling procedure	35
Figure S2	Boxplots showing appropriateness by location	36
Figure S3	Boxplots showing appropriateness by interaction partner	37
Figure S4	Within-person trajectories of psychological situation dimensions	38
Figure S5	Heatmap of simple-slope coefficients	39

Text S1: Sensitivity analysis for participant exclusion criterion

In the primary analyses, four participants who completed fewer than 30% of surveys (range: 1–6 of 21 surveys) were excluded. To assess whether this exclusion affected the results, all models were re-estimated including these participants (N = 146; 3,845 vocalisation observations). Results were virtually identical to the primary analyses (N = 142; 3,821 observations). Table Text S1.1 reports model fit indices for both samples. Across all models, R^2marginal differences were ≤ .001 and R^2conditional differences were ≤ .002, indicating that the exclusion had negligible impact on variance explained. All likelihood ratio tests remained highly significant (all ps < .001). Table Text S1.2 reports the H2c psychological dimension coefficients for both samples. All coefficients retained their direction, magnitude (maximum β difference = .002), and significance pattern, with the same four predictors significant (Positivity, Sociality, Formality, Power) and the same three non-significant (Negativity, Briskness, Novelty) in both analyses.

Table Text S1.1 Model fit comparison: Primary sample (N = 142) versus full sample including excluded participants (N = 146)

	***N* = 142**		***N* = 146**		**Difference**	
**Model**	**R²m**	**R²c**	**R²m**	**R²c**	**ΔR²m**	**ΔR²c**
Null (baseline)	.000	.211	.000	.210	.000	−.001
H1: Vocalisation type	.161	.369	.161	.368	.000	−.001
H2a: Location	.027	.244	.026	.243	−.001	−.001
H2b: Partner	.016	.236	.016	.235	.000	−.001
H2c: Psych. dimensions	.072	.255	.070	.253	.000	−.002
H3a: Vocal × Location	.175	.409	.175	.408	.000	−.001
H3b: Vocal × Partner	.177	.405	.177	.403	.000	−.002
H3c: Vocal × Psych. dim.	.233	.436	.234	.434	+.001	−.002

Note. R^2marginal = variance explained by fixed effects; R^2conditional = variance explained by both fixed and random effects. All likelihood ratio tests comparing each model to its null baseline remained significant at p < .001 in both samples.

Table Text S1.2 H2c psychological dimension coefficients: Primary sample (N = 142) versus full sample including excluded participants (N = 146)

	***N* = 142**			***N* = 146**			
**Predictor**	**β**	**SE**	**p**	**β**	**SE**	**p**	**Δβ**
Positivity	.154	.051	.002	.154	.051	.003	.000
Negativity	−.064	.050	.203	−.065	.050	.173	−.001
Sociality	.169	.031	<.001	.168	.031	<.001	−.001
Formality	−.190	.037	<.001	−.192	.037	<.001	−.002
Power	−.182	.037	<.001	−.180	.037	<.001	+.002
Briskness	.018	.035	.596	.018	.035	.420	.000

Novelty	−.002	.036	.955	.000	.036	.974	+.002

Note. β = standardised regression coefficient from multilevel models with person-mean centred predictors. Δβ = difference (*N* = 146 minus *N* = 142). All significance patterns are identical across samples: Positivity, Sociality, Formality, and Power are significant; Negativity, Briskness, and Novelty are non-significant.

Text S2 Experience sampling survey

The experience-sampling survey comprised four sections administered in fixed order.

Section 1: Location

Item 1. Location

Format: Single choice
Question: *"Where are you right now?"*

- ○ Home
- ○ Work
- ○ Campus
- ○ Bar
- ○ Party
- ○ Friend's house
- ○ Café/Restaurant
- ○ Gym
- ○ Library
- ○ Religious facility
- ○ Store/Mall
- ○ Other: _______________

Section 2: Interaction Partners

Item 2. Interaction partners

Format: Multiple select
Question: *"Who are you with right now?"*

- ☐ No one, alone
- ☐ Friends
- ☐ Family
- ☐ Significant other
- ☐ Roommates
- ☐ Classmates/Students/Co-workers
- ☐ Strangers
- ☐ Other: _______________

Section 3: Psychological Situation Characteristics

Item 3. Situation characteristics (7 sub-items)

Format: 5-point Likert scale per item
Question: *"Please rate your current context on the following characteristics."*

	1 = Not at all	2	3	4	5 = Extremely
This context is positive.	○	○	○	○	○
This context is negative.	○	○	○	○	○
Social interactions are possible or required.	○	○	○	○	○

This context is formal.	○	○	○	○	○
There is a clear power difference between people in this context.	○	○	○	○	○
This context is energetic or lively.	○	○	○	○	○
This context is unusual or unexpected.	○	○	○	○	○

Note. Items correspond to the dimensions Positivity, Negativity, Sociality, Formality, Power, Briskness, and Novelty, respectively. Dimension labels were not shown to participants.

Section 4: Vocalisations and Appropriateness

Item 4. Vocalisation observation

Format: Multiple select

Question: *"Which vocal expressions did you observe in this context? Select all that apply."*

- ☐ Cry
- ☐ Gasp
- ☐ Groan
- ☐ Grunt
- ☐ Laugh
- ☐ Moan
- ☐ Retch
- ☐ Growl
- ☐ Scream
- ☐ Sigh
- ☐ Other: _______________
- ☐ None observed

Note. If "None observed" was selected, the survey skipped directly to completion. If one or more vocalisations were selected, Item 5 appeared for each selected vocalisation.

Item 5. Perceived appropriateness (conditional; one rating per selected vocalisation)

Format: 9-point scale per vocalisation

Question: *"In general, how appropriate do you think most people would find expressing [vocalisation] in this context?"*

	1 Very inappropriate	2	3	4	5	6	7	8	9 Very appropriate
Cry	○	○	○	○	○	○	○	○	○
Gasp	○	○	○	○	○	○	○	○	○
Groan	○	○	○	○	○	○	○	○	○
Grunt	○	○	○	○	○	○	○	○	○
Laugh	○	○	○	○	○	○	○	○	○
Moan	○	○	○	○	○	○	○	○	○
Retch	○	○	○	○	○	○	○	○	○

Growl	○	○	○	○	○	○	○	○	○
Scream	○	○	○	○	○	○	○	○	○
Sigh	○	○	○	○	○	○	○	○	○

Note. Only vocalisations selected in Item 4 were displayed. The table above shows the full set for illustration. The phrasing "[vocalisation]" was replaced dynamically with each selected vocalisation type (e.g., "laughing," "screaming"). "Other" vocalisations, if selected, also received an appropriateness rating using the participant's free-text label.

Text S3 Location assessment

Location was assessed with the single-choice item *Where are you right now?* Response options were: Home, Work, Campus, Bar, Party, Friend's house, Café/Restaurant, Gym, Library, Religious facility, Store/Mall, or Other (free text).

Following Chung et al. [26], responses were recoded into four location categories based on the primary function of each setting:

- **Home** (selected "Home"): Private space for rest and unstructured time.
- **Work/School** (selected "Work" or "Campus"): Institutional settings with formal role expectations.
- **Social Place** (selected "Bar," "Party," or "Friend's house"): Venues where socialising with known others is the primary purpose of the visit.
- **Public Place** (selected "Café/Restaurant," "Gym," "Library," "Religious facility," or "Store/Mall"): Venues where people pursue individual activities such as eating, exercising, studying, shopping, or worship in shared spaces.

Free-text "Other" entries (n = 409) were reviewed and recoded into existing categories when possible. Uncodeable entries (n = 43) and entries lacking descriptions (n = 22) were retained as "Other" and excluded from analyses using the four-category scheme. Final distributions after recoding were: Home (n = 1,405), Work/School (n = 386), Social Place (n = 244), and Public Place (n = 570). Table Text S3.1 provides the full raw distributions and recoding decisions for all entries.

Table Text S3.1 Raw distributions of reported location entries and recoded categories

Response	Count (n)	% of total	Recoded Category
Home	1380	51.7	Home
Work	246	9.2	Work/School
Cafe, Restaurant	150	5.6	Public Place
Campus	126	4.7	Work/School
Friend's house	113	4.2	Social Place
Store/Mall	92	3.4	Public Place
Gym	66	2.5	Public Place
Library	27	1	Public Place
Bar	25	0.9	Social Place
Park	24	0.9	Social Place
Train	23	0.9	Public Place
Party	21	0.8	Social Place
Car	21	0.8	Public Place
Vacation	21	0.8	Other
Public transport	20	0.7	Public Place
Outside	18	0.7	Public Place
Bus	16	0.6	Public Place
Street	13	0.5	Public Place

Beach	12	0.4	Public Place
park	11	0.4	Social Place
Tram	9	0.3	Public Place
Hotel	8	0.3	Social Place
Cinema	7	0.3	Social Place
Airport	6	0.2	Public Place
metro	6	0.2	Public Place
Hospital	5	0.2	Work/School
car	5	0.2	Public Place
Religious facility	4	0.1	Public Place
Bus stop	4	0.1	Public Place
Outdoors	4	0.1	Other
Partner’s house	4	0.1	Home
plane	4	0.1	Public Place
train	4	0.1	Public Place
Metro	3	0.1	Public Place
Museum	3	0.1	Social Place
Road	3	0.1	Public Place
Zoo	3	0.1	Social Place
partner's house	3	0.1	Home
Apartment hunting	2	0.1	Public Place
Bank	2	0.1	Public Place
Boyfriends house	2	0.1	Other
Festival	2	0.1	Social Place
Girlfriend,Äôs house	2	0.1	Other
Market	2	0.1	Social Place
Metro stop	2	0.1	Public Place
Office	2	0.1	Work/School
Supermarket	2	0.1	Public Place
Train station	2	0.1	Public Place
Transport	2	0.1	Public Place
beach	2	0.1	Public Place
in my car	2	0.1	Public Place
road	2	0.1	Public Place
street	2	0.1	Public Place
Antic market	1	0	Social Place
Art Gallery	1	0	Social Place
At a workshop	1	0	Work/School
At my boyfriends place	1	0	Home
At my grandparents	1	0	Home
At my partner,Äôs house	1	0	Home
Bf house	1	0	Home
Bike repair store	1	0	Public Place

Bioscoop	1	0	Social Place
Boyfriends‚Äô house	1	0	Other
Bus station	1	0	Public Place
Bus to work	1	0	Other
Busstop	1	0	Public Place
Cafe	1	0	Public Place
Changing room	1	0	Other
City for kingsday	1	0	Social Place
City tour	1	0	Other
Class	1	0	Other
Climbing gym	1	0	Social Place
Clinic	1	0	Work/School
Coffee bar of a friend	1	0	Social Place
Concert	1	0	Other
Construction Side	1	0	Social Place
De pont	1	0	Social Place
Dentist	1	0	Work/School
Dentist's	1	0	Work/School
Dining room	1	0	Home
Drivers lesson	1	0	Work/School
Drivers lesson (being brought home)	1	0	Work/School
Ferry	1	0	Public Place
Ferry boat	1	0	Other
Festival (by the creek)	1	0	Social Place
Forest	1	0	Social Place
Garden	1	0	Social Place
Gas station	1	0	Public Place
Grandmas house	1	0	Home
Hairdresser	1	0	Public Place
Hairdresser	1	0	Public Place
Holiday parc	1	0	Home
Home / Balcony	1	0	Home
Hostel	1	0	Home
House of a family member	1	0	Home
I work in an old book bazaar	1	0	Work/School
In a bus	1	0	Public Place
In bed	1	0	Home
In car	1	0	Other
In the car	1	0	Public Place
In the carpark	1	0	Public Place
Just woke up	1	0	Home
Laundry	1	0	Other
Metro station	1	0	Public Place

Mosque	1	0	Social Place
Movies	1	0	Social Place
My family’s village vacation house	1	0	Home
My grandparents home in Italy	1	0	Home
My grandparents,Äô home	1	0	Home
My other house in the mountains	1	0	Home
My parents village house	1	0	Home
My village	1	0	Home
My village house	1	0	Home
On a boat in the Amsterdam canals for kingsday	1	0	Social Place
On a car ride	1	0	Public Place
On a walk	1	0	Public Place
On a walk outside	1	0	Public Place
On an airplane	1	0	Public Place
On the Road	1	0	Other
On the ferry	1	0	Public Place
On the street	1	0	Public Place
Online Teams meeting	1	0	Work/School
Outside walking in the city	1	0	Public Place
Parking lot	1	0	Public Place
Plane	1	0	Public Place
Public Transport	1	0	Public Place
Public Transport (metro)	1	0	Public Place
Public Transportation	1	0	Public Place
Public facility	1	0	Public Place
Public transportation	1	0	Public Place
Public transportation (bus)	1	0	Public Place
Racing course	1	0	Social Place
Right outside my building	1	0	Public Place
Road to Home	1	0	Public Place
Rowing course	1	0	Social Place
Sailing	1	0	Social Place
Shared living room facility at my appartement complex	1	0	Home
Spa	1	0	Other
Sporting facility (hockey)	1	0	Social Place
Still on the boat in Amsterdam	1	0	Social Place
Swimming pool	1	0	Social Place
Tattoo studio	1	0	Social Place
The house im staying at from work	1	0	Home
Theater	1	0	Social Place
Travelling	1	0	Public Place
Ttain	1	0	Public Place

Viewing	1	0	Public Place
Volleyball hall	1	0	Other
Walking in Amsterdam	1	0	Public Place
Walking in a Neighborhood	1	0	Public Place
Walking in the street	1	0	Public Place
Walking on the street	1	0	Public Place
Walking through the city center	1	0	Public Place
Was on a walk at the time of the notification	1	0	Other
Was unable to fill in the survey	1	0	Other
With my boyfriend	1	0	Other
Zoom with friend	1	0	Other
bus	1	0	Public Place
business conference	1	0	Work/School
busy street	1	0	Public Place
city center	1	0	Public Place
city streets	1	0	Public Place
dressing room at gym	1	0	Public Place
hospital	1	0	Work/School
in airplane	1	0	Public Place
movies	1	0	Social Place
on the street	1	0	Public Place
online meeting	1	0	Other
outside	1	0	Public Place
outside in front of my house	1	0	Public Place
relatives	1	0	Social Place
house	1	0	Home
shopping street	1	0	Social Place
tailor	1	0	Social Place
tram	1	0	Public Place
vacation	1	0	Other
walking to metro	1	0	Public Place
with goats at the park	1	0	Social Place

Note. Locations were collapsed to higher-order categories. Free-text Other entries were hand-coded when interpretable; remaining Other were retained as “Other” and excluded from category-specific analyses.

Text S4. Sensitivity analysis: excluding multiple interaction partner observations

Overview. In the primary analysis, each interaction partner selection was treated as a separate observation (see Methods). Of 2,670 surveys, 275 (10.3%) involved multiple partner categories. In this sensitivity analysis, these multi-partner observations were excluded entirely to test whether results are robust when only unambiguous, single-partner observations are retained. The filtered dataset contained 3,179 vocalisation observations from 2,395 surveys.

Model Fit

All seven hypothesis models remained significant in likelihood ratio tests against the null model (all ps < .001). Table Text S4.1 compares model fit indices between the primary and single-partner analyses.

Table Text S4.1 Model fit comparison

**Model**	**R^2m (Orig)**	**R^2m (Single)**	**ΔR^2m**	**R^2c (Orig)**	**R^2c (Single)**	**ΔR^2c**
H1: Vocalisation type	.161	.151	−.010	.369	.366	−.003
H2a: Location	.027	.028	+.000	.240	.247	+.007
H2b: Partner	.017	.029	+.012	.240	.259	+.019
H2c: Psych. dimensions	.072	.077	+.005	.264	.275	+.012
H3a: Vocal × Location	.195	.187	−.007	.422	.416	−.006
H3b: Vocal × Partner	.186	.186	−.001	.413	.413	+.000
H3c: Vocal × Psych.	.252	.248	−.004	.454	.450	−.004

Note. R^2m = marginal R^2 (fixed effects); R^2c = conditional R^2 (fixed + random effects). All R^2 differences are within |.015|.

Psychological Dimensions (H2c)

All significant predictors in the primary analysis remained significant, and all non-significant predictors remained non-significant. The direction and relative magnitude of all effects were preserved.

Table Text S4.2 H2c coefficient comparison

**Predictor**	**β (Orig)**	**p (Orig)**	**β (Single)**	**p (Single)**	**Δβ**	**Sig. change?**
Positivity	.154	.002	.200	<.001	+.046	No
Negativity	−.064	.203	−.031	.576	+.033	No
Sociality	.169	<.001	.186	<.001	+.017	No
Formality	−.190	<.001	−.187	<.001	+.003	No

Power	−.182	<.001	−.152	<.001	+.030	No
Briskness	.018	.596	.020	.596	+.002	No
Novelty	−.002	.955	+.025	.524	+.027	No

Fixed Effects Comparison

Across all seven models, 138 of 146 fixed effects (95%) maintained the same significance status. Eight effects changed significance at the $p < .05$ threshold, all near the boundary.

Table Text S4.3 Effects that changed significance.

Effects that lost significance (primary sig., single-partner *n*.s.)

**Model**	**Predictor**	**Est. (Orig)**	**p (Orig)**	**Est. (Single)**	**p (Single)**
H3a	Location: Public Place	−0.496	.038	−0.365	.152
H3a	Groan × Social Place	−1.273	.007	−0.910	.100
H3a	Grunt × Social Place	−1.118	.048	−0.676	.331
H3a	Sigh × Social Place	−0.862	.026	−0.705	.130
H3c	Grunt × Sociality	−0.362	.006	−0.276	.055

Effects that gained significance (primary *n*.s., single-partner sig.)

**Model**	**Predictor**	**Est. (Orig)**	**p (Orig)**	**Est. (Single)**	**p (Single)**
H2a	Social Place	0.151	.213	0.287	.033
H3a	Scream × Work/School	0.788	.068	1.623	.001
H3c	Laugh × Power	−0.173	.129	−0.273	.028

Note. Significance changes are concentrated in H3a interaction terms involving Social Place, the context most affected by multi-partner filtering. Critically, all H2b and H3b partner effects retained identical significance patterns.

Conclusion

The primary analysis is robust to the exclusion of multi-partner observations. All models remained significant, all H2c patterns were preserved, and 95% of fixed effects maintained the same significance status. The partner models (H2b, H3b), which are most directly relevant to the interaction partner coding, showed identical significance patterns.

Text S5 Interaction partner assessment

Interaction partners were assessed with the multiple-select item *Who are you with right now?* Response options were: Alone, Friends, Family, Significant other, Roommates, Classmates/Students/Co-workers, Strangers, or Other (free text).

Following Chung et al. [26], responses were recoded into four categories based on relationship closeness and social function:

- **Alone** (selected "Alone"): No interaction partner present.
- **Close Ties** (selected "Friends," "Family," "Roommates," or "Significant other"): Individuals with whom the participant shares an established, intimate relationship.
- **Weak Ties** (selected "Classmates/Students/Co-workers"): Individuals known through institutional roles but without close personal relationships.
- **Strangers** (selected "Strangers"): Unfamiliar individuals with no prior relationship.

Because the item allowed multiple selections, participants who selected more than one interaction partner contributed multiple entries to the dataset (e.g., a participant selecting both "Friends" and "Strangers" contributed one observation to Close Ties and one to Strangers).

Free-text "Other" entries (n = 31) were reviewed and recoded into existing categories when possible. Uncodeable entries (n = 1) and entries lacking descriptions (n = 4) were retained as "Other" and excluded from analyses using the four-category scheme. Final distributions after recoding were: Alone (n = 933), Close Ties (n = 1,288), Weak Ties (n = 318), and Strangers (n = 371). Table Text S5.1 provides the full raw distributions.

Table Text S5.1 Raw distributions of reported interaction partners and recoded categories

**Response**	**Count (n)**	**% of total**	**Recoded Category**
alone	938	35.1	alone
Family	490	18.4	Family
Friends	426	16	Friends
Strangers	376	14.1	Strangers
Classmates/Students/Co-workers	320	12	Classmates/Students/Co-workers
Significant Other	303	11.3	Significant Other
Roommates	71	2.7	Roommates
Other	20	0.7	Other
Client	2	0.1	Weak Ties
Collega	2	0.1	Weak Ties
Grandmother	2	0.1	Close Ties
Mother	2	0.1	Close Ties
Therapist	2	0.1	Weak Ties
Airport	1	0	Uncategorised
Church family	1	0	Close Ties
Classmates, teacher and a client in an online meeting	1	0	Weak Ties

Doctor	1	0	Weak Ties
Driver instructor and other student	1	0	Weak Ties
Driving instructor	1	0	Weak Ties
Friend	1	0	Close Ties
Kid	1	0	Close Ties
Museum	1	0	Uncategorised
My dad	1	0	Close Ties
My dog	1	0	Uncategorised
My mom	1	0	Close Ties
On the phone with my boss	1	0	Weak Ties
Outside	1	0	Uncategorised
Park	1	0	Uncategorised
Partner	1	0	Close Ties
Street	1	0	Uncategorised
Teammates	1	0	Other
Train	1	0	Uncategorised
driving instructor	1	0	Weak Ties
my little sisters and their friends	1	0	Close Ties

Note. Interaction partners were collapsed to four categories. Free-text Other entries were hand-coded to Close ties or Weak ties when interpretable (values shown in table); remaining Other were retained as “Other” and excluded from category-specific analyses.

Text S6. Vocalisation assessment: item wording, response options, and exclusions

Vocalisations were assessed with the multiple-select item *Which vocal expressions did you observe in this context? Select all that apply.* Participants reported vocalisations they had observed in the current context, including both their own and others' vocalisations. Response options were: Cry, Gasp, Groan, Grunt, Laugh, Moan, Retch, Growl, Scream, Sigh, Other (with free text), and None observed.

This set of vocalisations follows established taxonomies derived from previous research [21,29]. The selection was based on semantic grouping through naming tasks that identified distinct vocal sounds, with minor modifications including merging acoustically similar categories (e.g., shriek/scream). Multiple selections were permitted to capture co-occurring vocalisations.

Free-text "Other" entries (*n* = 229) were excluded from main analyses but are provided in Table Text S6.1 for exploratory purposes, along with "Other" responses lacking descriptions (*n* = 4). Survey responses where participants selected only "None observed" (*n* = 625) were excluded from vocalisation analyses, as they contained no vocalisation data to analyse. Table Text S6.2 provides the raw observation counts for each vocalisation type.

Table Text S6.1 Free-text “Other” vocalisations and their counts

**Category**	***n***
Speech (e.g., speech prosody, talking sounds, chattering)	49
Yawn	45
Cough/Sneeze/Throat clear	19
Snoring	10
Humming	9
Interjection (humph/harrumph/scoff/tsk/puff/huff)	9
Singing/Croon	8
Music/TV/Media	8
Chewing/Eating	7
Shout/Yell/Scream	7
Breathing	6
Environmental/Mechanical	6
Snort	5
Laughter/Chuckle	5
Animal/Bird	5
Whisper	4
Affect burst (aww/aah)	3
Vocal filler (hmm/uh/uhu)	3
Sniff/Sniffle	3
Whistling	1
Hiccup	1
Burp	1

Category	*n*
Sigh	1
Label/Other (non-sound)	10
Other/Uncategorised	4

Note. Free-text labels were normalised (lower-cased, minor typos fixed) and collapsed into categories (e.g., yawn/yawning → Yawn; chattering/speech prosody → Speech). Strings were coded to the dominant human-vocal element.

Table Text S6.2. Raw observation counts and percentages for vocalisation types

Category	Count (N)	% of all observations
Laugh	1222	32.00%
Sigh	935	24.50%
Gasp	369	9.70%
Groan	309	8.10%
Scream	302	7.90%
Grunt	233	6.10%
Moan	198	5.20%
Cry	141	3.70%
Growl	65	1.70%
Retch	47	1.20%

Note. Percentages are pooled across all prompts (prompt-weighted): % = (prompts with category ÷ all prompts) × 100. These prompt-weighted percentages differ from the participant-mean percentages reported in Table S2, which first compute within-person percentages and then average across participants.

Text S7 Robustness check: within-between decomposition of categorical predictors

To confirm that the location (H2a) and interaction partner (H2b) effects represent within-person situational variation rather than between-person differences in context exposure, contextual models were estimated that included grand-mean centred person-level proportions alongside the occasion-level categorical predictors (see main text Statistical Analysis section). Tables Text S7.1 and Text S7.2 report the results.

Table Text S7.1 Within-person and between-person coefficient estimates from contextual models

Model	Predictor	Effect type	β	SE	t	p
**H2a: Location**	Public Place (vs. Home)	Within	−0.659	0.079	−8.33	<.001
	Social Place (vs. Home)	Within	+0.121	0.105	1.16	.246
	Work/School (vs. Home)	Within	−0.669	0.095	−7.01	<.001
	Prop. Home (GM-centred)	Between	+0.412	0.732	0.56	.573
	Prop. Work/School (GM-centred)	Between	+0.178	0.908	0.20	.845
	Prop. Social Place (GM-centred)	Between	+0.849	0.626	1.36	.174
**H2b: Partner**	Close ties (vs. Alone)	Within	+0.280	0.093	2.99	.003
	Strangers (vs. Alone)	Within	−0.381	0.109	−3.51	<.001
	Weak ties (vs. Alone)	Within	−0.296	0.118	−2.51	.012
	Prop. Alone (GM-centred)	Between	+0.196	0.454	0.43	.667
	Prop. Close ties (GM-centred)	Between	+1.761	0.902	1.95	.051
	Prop. Weak ties (GM-centred)	Between	+1.058	0.720	1.47	.142

Note. Within-person effects represent changes in appropriateness when the same participant moves between contexts. Between-person effects represent associations between the proportion of surveys a participant spent in each context (grand-mean centred) and their overall appropriateness ratings. Reference categories: Home (H2a), Alone (H2b). None of the between-person effects were statistically significant.

Table Text S7.2 Model fit comparison: original models vs. within-between contextual models

	**Original model**		**Within-between model**	
**Model**	$R^2_{marginal}$	$R^2_{conditional}$	$R^2_{marginal}$	$R^2_{conditional}$
**H2a: Location**	.027	.240	.026	.242
**H2b: Partner**	.017	.240	.020	.238

Note. $R^2_{marginal}$ = variance explained by fixed effects; $R^2_{conditional}$ = variance explained by both fixed and random effects. The negligible change in R^2 values confirms that between-person differences in context exposure explain virtually no additional variance beyond within-person situational effects

Table S1 Distribution of participant nationalities

**Nationality**	***n***	**%**
Netherlands	42	29.6
India	15	10.6
Turkey	14	9.9
Germany	8	5.6
Finland	6	4.2
Italy	5	3.5
Iran	4	2.8
Romania	4	2.8
Bulgaria	3	2.1
China	3	2.1
Greece	3	2.1
Germany, United Kingdom	2	1.4
Indonesia	2	1.4
Russian Federation	2	1.4
Serbia	2	1.4
Spain	2	1.4
United States of America	2	1.4
Vietnam	2	1.4
Austria	1	0.7
Brazil	1	0.7
Colombia	1	0.7
Croatia, Serbia	1	0.7
Czech Republic	1	0.7
France	1	0.7
Ireland	1	0.7
Italy, Spain	1	0.7
Lithuania	1	0.7
Mauritius	1	0.7
Mexico	1	0.7
Pakistan	1	0.7
Peru	1	0.7
Poland	1	0.7
Portugal	1	0.7
Slovakia	1	0.7
South Africa	1	0.7
South Korea	1	0.7
Sweden	1	0.7
Thailand	1	0.7
Uzbekistan	1	0.7

Table S2 Descriptive results of the main variables

Variable	M	SD	Min	Max	ICC
**Location**					
Home	59.95	20.29	0	100	–
Public Place	16.35	12.97	0	50	–
Social Place	7.41	9.96	0	52.63	–
Work/School	16.29	16.47	0	100	–
**Interaction partner**					
Alone	33.17	21.45	0	100	–
Close ties	43.16	23.54	0	100	–
Strangers	12.49	10.4	0	46.15	–
Weak ties	11.18	13.12	0	90.48	–
**Vocalisation**					
Cry	4.46	7.19	0	42.86	–
Gasp	12.71	16.01	0	100	
Groan	12.05	12.82	0	61.11	–
Growl	2.37	5.14	0	42.11	–
Grunt	8.23	10.45	0	58.33	–
Laugh	44.65	19.35	0	85.71	–
Moan	7.66	11.87	0	66.67	–
Retch	1.61	5.01	0	42.11	–
Scream	10.43	12.53	0	75	–
Sigh	34.29	22.81	0	85.71	–
**Psychological situation dimensions**					
Positivity	3.70	1.00	1	5	.213
Negativity	1.63	0.90	1	5	.228
Sociality	3.01	1.48	1	5	.204
Formality	1.64	1.06	1	5	.227
Power	1.67	1.04	1	5	.229
Novelty	1.51	0.87	1	5	.201
Briskness	2.86	1.23	1	5	.195

Note. Categorical predictors are reported as participant-mean percentages: for each participant, the percentage of their prompts in which a category occurred was computed and then averaged across participants. For example, “Home = 59.95” means that, on average, participants were at home for 59.95% of their prompts. ICC = Intraclass correlation.

Table S3 Model fit indices and comparisons across hypothesis models

Model	*N* obs	AIC	BIC	R^2 marginal	R^2 conditional	ΔAIC	χ^2	*p*
Null (baseline)	3,821	15,755.6	15,774.3	.000	.211	—	—	—
H1: Vocalisation type	3,821	14,977.9	15,052.8	.161	.369	−777.7	816.54	<.001
H2a: Location	3,131	12,812.8	12,849.0	.027	.244	−76.2	90.58	<.001
H2b: Partner	3,131	12,854.7	12,897.0	.016	.236	−34.2	50.15	<.001
H2c: Psych. dimensions	3,710	15,090.6	15,196.4	.072	.264	−205.9	233.88	<.001
H3a: Vocal × Location	2,936	11,354.2	11,533.8	.175	.409	−622.9	712.32	<.001
H3b: Vocal × Partner	2,936	11,345.7	11,567.1	.177	.405	−631.4	719.37	<.001
H3c: Vocal × Psych. dim.	2,936	11,314.7	11,661.9	.233	.436	−662.4	934.15	<.001

Note. All models include random intercepts for participants. ΔAIC = difference in AIC relative to a null model with random intercepts only fitted to the same subset of observations. χ^2 = likelihood ratio test statistic comparing each model to its corresponding null baseline. R^2marginal = variance explained by fixed effects; R^2conditional = variance explained by both fixed and random effects. For the null model, R^2conditional equals the ICC (.211). H2 and H3 models have smaller *N*_obs because analyses were restricted to observations with complete situational data (H2) and to vocalisation types with ≥20 observations per cell (H3). The η^2 values reported in the main text for H3a–H3c refer specifically to the interaction terms, whereas R^2marginal reflects all fixed effects in the model.

Table S4 Pairwise comparisons of appropriateness ratings across vocalisation types

Vocalisation A	Vocalisation B	Mean Difference	d	p-value
Cry	Gasp	-1.12	-0.68	<.001
Cry	Groan	-0.76	-0.46	<.001
Cry	Growl	-0.11	-0.07	1
Cry	Grunt	-0.64	-0.39	0.014
Cry	Laugh	-2.15	-1.31	<.001
Cry	Moan	-0.58	-0.35	0.063
Cry	Retch	0.64	0.39	0.412
Cry	Scream	0.51	0.31	0.091
Cry	Sigh	-1.26	-0.77	<.001
Gasp	Groan	0.36	0.22	0.166
Gasp	Growl	1.01	0.61	<.001
Gasp	Grunt	0.48	0.29	0.028
Gasp	Laugh	-1.03	-0.63	<.001
Gasp	Moan	0.54	0.33	0.013
Gasp	Retch	1.76	1.07	<.001
Gasp	Scream	1.62	0.99	<.001
Gasp	Sigh	-0.15	-0.09	0.928
Groan	Growl	0.65	0.4	0.126
Groan	Grunt	0.12	0.07	0.998
Groan	Laugh	-1.39	-0.84	<.001
Groan	Moan	0.18	0.11	0.977
Groan	Retch	1.4	0.85	<.001
Groan	Scream	1.27	0.77	<.001
Groan	Sigh	-0.5	-0.31	<.001
Growl	Grunt	-0.53	-0.32	0.424
Growl	Laugh	-2.04	-1.24	<.001
Growl	Moan	-0.47	-0.29	0.627
Growl	Retch	0.75	0.46	0.353
Growl	Scream	0.62	0.38	0.17
Growl	Sigh	-1.15	-0.7	<.001
Grunt	Laugh	-1.51	-0.92	<.001
Grunt	Moan	0.06	0.04	1
Grunt	Retch	1.28	0.78	<.001
Grunt	Scream	1.15	0.7	<.001
Grunt	Sigh	-0.62	-0.38	<.001
Laugh	Moan	1.57	0.95	<.001
Laugh	Retch	2.79	1.7	<.001
Laugh	Scream	2.66	1.62	<.001

Laugh	Sigh	0.88	0.54	<.001
Moan	Retch	1.22	0.74	<.001
Moan	Scream	1.09	0.66	<.001
Moan	Sigh	-0.68	-0.42	<.001
Retch	Scream	-0.13	-0.08	1
Retch	Sigh	-1.9	-1.16	<.001
Scream	Sigh	-1.77	-1.08	<.001

Note. Tukey-adjusted pairwise comparisons between all vocalisation types.

Table S5 Pairwise comparisons of appropriateness across locations

Location A	Location B	Mean Difference	d	p-value
Home	Public Place	0.65	0.36	<.001
Home	Social Place	−0.13	−0.07	.590
Home	Work/School	0.65	0.36	<.001
Public Place	Social Place	−0.78	−0.43	<.001
Public Place	Work/School	0.00	0.00	1.000
Social Place	Work/School	0.78	0.43	<.001

Note. Tukey-adjusted pairwise comparisons between location categories.

Table S6 Pairwise comparisons of appropriateness across interaction partners

Partner A	Partner B	Mean Difference	d	p-value
Alone	Close ties	-0.29	-0.16	0.01
Alone	Strangers	0.35	0.2	0.006
Alone	Weak ties	0.27	0.15	0.103
Close ties	Strangers	0.64	0.36	<.001
Close ties	Weak ties	0.55	0.31	<.001
Strangers	Weak ties	-0.09	-0.05	0.846

Note. Tukey-adjusted pairwise comparisons between interaction partner categories.

Table S7 Vocalisation × Location interactions

contrast	vocalisation	estimate	SE	df	t.ratio	p.value
Home - Public Place	Gasp	0.41	0.21	3353.13	2.00	0.14
Home - Social Place	Gasp	-0.42	0.26	3350.25	-1.59	0.22
Home - (Work/School)	Gasp	0.66	0.26	3346.92	2.50	0.05
Public Place - Social Place	Gasp	-0.83	0.27	3323.96	-3.12	0.01
Public Place - (Work/School)	Gasp	0.25	0.27	3326.57	0.92	0.36
Social Place - (Work/School)	Gasp	1.08	0.31	3319.49	3.46	<0.01
Home - Public Place	Groan	0.76	0.22	3342.01	3.43	<0.01
Home - Social Place	Groan	1.17	0.31	3328.47	3.75	<0.01
Home - (Work/School)	Groan	0.56	0.27	3339.43	2.03	0.17
Public Place - Social Place	Groan	0.41	0.33	3322.56	1.26	0.41
Public Place - (Work/School)	Groan	-0.20	0.29	3340.84	-0.69	0.49
Social Place - (Work/School)	Groan	-0.62	0.37	3326.00	-1.69	0.28
Home - Public Place	Grunt	0.50	0.25	3325.48	2.04	0.25
Home - Social Place	Grunt	0.64	0.39	3319.50	1.66	0.39
Home - (Work/School)	Grunt	0.65	0.32	3334.80	2.02	0.25
Public Place - Social Place	Grunt	0.14	0.38	3314.87	0.37	1.00
Public Place - (Work/School)	Grunt	0.15	0.32	3333.83	0.46	1.00
Social Place - (Work/School)	Grunt	0.00	0.44	3323.21	0.01	1.00
Home - Public Place	Laugh	0.45	0.12	3378.35	3.90	<0.01
Home - Social Place	Laugh	-0.56	0.14	3360.37	-3.95	<0.01
Home - (Work/School)	Laugh	1.05	0.14	3380.48	7.78	<0.01
Public Place - Social Place	Laugh	-1.02	0.14	3340.63	-7.03	<0.01
Public Place - (Work/School)	Laugh	0.60	0.14	3375.97	4.31	<0.01
Social Place - (Work/School)	Laugh	1.62	0.16	3357.35	10.04	<0.01
Home - Public Place	Moan	1.15	0.27	3327.21	4.20	<0.01
Home - Social Place	Moan	0.53	0.40	3349.22	1.34	0.59
Home - (Work/School)	Moan	1.06	0.40	3349.89	2.64	0.04
Public Place - Social Place	Moan	-0.62	0.43	3345.37	-1.45	0.59

Public Place - (Work/School)	Moan	-0.09	0.43	3344.57	-0.20	0.84
Social Place - (Work/School)	Moan	0.53	0.52	3354.94	1.02	0.61
Home - Public Place	Scream	0.89	0.23	3368.73	3.91	<0.01
Home - Social Place	Scream	-0.91	0.26	3347.20	-3.45	<0.01
Home - (Work/School)	Scream	-0.11	0.32	3368.84	-0.36	0.72
Public Place - Social Place	Scream	-1.81	0.26	3318.80	-6.94	<0.01
Public Place - (Work/School)	Scream	-1.01	0.32	3357.95	-3.18	<0.01
Social Place - (Work/School)	Scream	0.80	0.34	3346.51	2.32	0.04
Home - Public Place	Sigh	0.44	0.13	3343.68	3.23	0.01
Home - Social Place	Sigh	0.58	0.19	3353.18	3.03	0.01
Home - (Work/School)	Sigh	0.62	0.14	3359.47	4.32	<0.01
Public Place - Social Place	Sigh	0.15	0.21	3342.62	0.72	0.95
Public Place - (Work/School)	Sigh	0.19	0.16	3349.00	1.14	0.76
Social Place - (Work/School)	Sigh	0.04	0.21	3346.16	0.19	0.95

Note. Pairwise contrasts comparing locations within each vocalisation type. p-values are Holm-Bonferroni corrected.

Table S8 Vocalisation × Interaction partner interactions

contrast	vocalisation	estimate	SE	df	t.ratio	p.value
Alone - Close ties	Gasp	0.11	0.26	4071.85	0.44	1.00
Alone - Strangers	Gasp	0.23	0.29	4066.82	0.79	1.00
Alone - Weak ties	Gasp	0.59	0.33	4054.78	1.78	0.38
Close ties - Strangers	Gasp	0.12	0.21	3968.55	0.58	1.00
Close ties - Weak ties	Gasp	0.47	0.25	3960.04	1.86	0.38
Strangers - Weak ties	Gasp	0.35	0.29	3960.85	1.21	0.90
Alone - Close ties	Groan	0.49	0.24	3975.73	2.08	0.19
Alone - Strangers	Groan	0.65	0.29	3977.44	2.25	0.15
Alone - Weak ties	Groan	0.61	0.34	3958.74	1.79	0.30
Close ties - Strangers	Groan	0.15	0.23	3973.16	0.67	1.00
Close ties - Weak ties	Groan	0.11	0.29	3959.98	0.38	1.00
Strangers - Weak ties	Groan	-0.04	0.33	3960.51	-0.12	1.00
Alone - Close ties	Grunt	0.72	0.28	3964.15	2.56	0.06
Alone - Strangers	Grunt	0.47	0.32	3968.35	1.46	0.58
Alone - Weak ties	Grunt	0.63	0.37	3972.33	1.70	0.45
Close ties - Strangers	Grunt	-0.25	0.24	3957.92	-1.04	0.90
Close ties - Weak ties	Grunt	-0.09	0.31	3962.24	-0.28	1.00
Strangers - Weak ties	Grunt	0.17	0.34	3958.41	0.48	1.00
Alone - Close ties	Laugh	-1.22	0.16	4068.90	-7.59	0.00
Alone - Strangers	Laugh	-0.30	0.18	4047.42	-1.65	0.20
Alone - Weak ties	Laugh	0.04	0.19	4057.04	0.21	0.83
Close ties - Strangers	Laugh	0.92	0.12	4015.87	7.45	<0.01
Close ties - Weak ties	Laugh	1.26	0.13	4016.41	9.67	<0.01

Strangers - Weak ties	Laugh	0.34	0.16	3993.09	2.17	0.09
Alone - Close ties	Moan	0.20	0.31	3975.33	0.64	1.00
Alone - Strangers	Moan	0.85	0.37	3969.95	2.26	0.14
Alone - Weak ties	Moan	0.66	0.44	3981.42	1.51	0.53
Close ties - Strangers	Moan	0.65	0.29	3958.14	2.24	0.14
Close ties - Weak ties	Moan	0.46	0.37	3980.72	1.26	0.63
Strangers - Weak ties	Moan	-0.19	0.43	3972.43	-0.44	1.00
Alone - Close ties	Scream	-1.12	0.28	4020.54	-3.98	<0.01
Alone - Strangers	Scream	-0.12	0.32	4009.29	-0.38	1.00
Alone - Weak ties	Scream	-0.94	0.38	4022.06	-2.48	0.05
Close ties - Strangers	Scream	1.00	0.23	3969.85	4.38	0.00
Close ties - Weak ties	Scream	0.18	0.30	4005.17	0.59	1.00
Strangers - Weak ties	Scream	-0.82	0.34	3990.94	-2.40	0.05
Alone - Close ties	Sigh	0.43	0.13	4008.12	3.21	0.005
Alone - Strangers	Sigh	0.64	0.17	3990.07	3.69	0.001
Alone - Weak ties	Sigh	0.68	0.17	3998.20	3.96	0.00
Close ties - Strangers	Sigh	0.21	0.15	3982.41	1.40	0.32
Close ties - Weak ties	Sigh	0.25	0.15	3994.68	1.70	0.27
Strangers - Weak ties	Sigh	0.04	0.18	3974.14	0.24	0.81

Note. Pairwise contrasts comparing interaction partners within each vocalisation type. p-values are Holm-Bonferroni corrected.

Table S9 Simple slopes of psychological situation dimensions by vocalisation

characteristic	vocalisation	slope	se	t_ratio	df	p_raw	p_holm	ci_lower	ci_upper
Positivity	Gasp	0.32	0.14	2.30	2878.83	0.02	0.11	-0.05	0.68
Positivity	Groan	-0.19	0.14	-1.37	2793.25	0.17	0.68	-0.57	0.18
Positivity	Grunt	0.01	0.18	0.03	2783.88	0.98	1.00	-0.48	0.49
Positivity	Laugh	0.26	0.08	3.38	2866.98	<.001	0.005	0.05	0.46
Positivity	Moan	0.19	0.17	1.10	2797.95	0.27	0.82	-0.28	0.66
Positivity	Scream	0.39	0.14	2.82	2810.19	0.01	0.03	0.02	0.76
Positivity	Sigh	-0.06	0.08	-0.68	2825.67	0.50	1.00	-0.28	0.17
Negativity	Gasp	0.03	0.16	0.19	2801.01	0.85	1.00	-0.39	0.45
Negativity	Groan	0.01	0.13	0.11	2793.82	0.91	1.00	-0.34	0.37
Negativity	Grunt	-0.13	0.17	-0.79	2777.95	0.43	1.00	-0.59	0.33
Negativity	Laugh	-0.07	0.09	-0.75	2861.36	0.45	1.00	-0.30	0.17
Negativity	Moan	0.09	0.17	0.50	2784.97	0.62	1.00	-0.38	0.56
Negativity	Scream	0.13	0.15	0.88	2802.19	0.38	1.00	-0.28	0.54
Negativity	Sigh	-0.03	0.08	-0.34	2842.47	0.73	1.00	-0.24	0.19
Sociality	Gasp	0.01	0.08	0.09	2861.72	0.93	1.00	-0.22	0.23
Sociality	Groan	0.06	0.08	0.67	2789.09	0.50	1.00	-0.17	0.28
Sociality	Grunt	-0.36	0.11	-3.39	2784.43	<.001	0.004	-0.64	-0.07
Sociality	Laugh	0.39	0.05	7.71	2844.04	<.001	<.001	0.26	0.53
Sociality	Moan	0.01	0.11	0.08	2785.66	0.94	1.00	-0.29	0.31
Sociality	Scream	0.30	0.10	3.00	2832.52	0.00	0.014	0.03	0.56
Sociality	Sigh	-0.05	0.05	-1.00	2804.55	0.32	1.00	-0.19	0.09
Formality	Gasp	-0.22	0.10	-2.21	2796.06	0.03	0.11	-0.48	0.05
Formality	Groan	-0.13	0.10	-1.32	2804.85	0.19	0.49	-0.40	0.14
Formality	Grunt	-0.16	0.11	-1.40	2794.92	0.16	0.49	-0.46	0.15
Formality	Laugh	-0.32	0.06	-5.64	2862.27	<.001	<.001	-0.47	-0.17

Formality	Moan	-0.40	0.15	-2.67	2788.21	0.01	0.05	-0.81	0.00
Formality	Scream	-0.32	0.13	-2.51	2798.91	0.01	0.06	-0.67	0.02
Formality	Sigh	-0.08	0.06	-1.36	2829.14	0.17	0.49	-0.25	0.08
Power	Gasp	-0.02	0.10	-0.24	2786.15	0.81	1.00	-0.30	0.25
Power	Groan	-0.10	0.10	-1.01	2804.56	0.31	1.00	-0.36	0.17
Power	Grunt	0.07	0.12	0.61	2778.30	0.54	1.00	-0.25	0.40
Power	Laugh	-0.20	0.06	-3.48	2850.08	<.001	0.004	-0.35	-0.05
Power	Moan	-0.07	0.12	-0.53	2788.66	0.60	1.00	-0.40	0.27
Power	Scream	-0.06	0.10	-0.56	2828.96	0.58	1.00	-0.33	0.22
Power	Sigh	-0.17	0.07	-2.52	2803.25	0.01	0.07	-0.35	0.01
Briskness	Gasp	-0.01	0.10	-0.07	2862.44	0.94	1.00	-0.26	0.25
Briskness	Groan	-0.04	0.09	-0.41	2797.53	0.68	1.00	-0.27	0.20
Briskness	Grunt	0.22	0.12	1.84	2791.53	0.07	0.33	-0.10	0.54
Briskness	Laugh	0.17	0.06	3.03	2834.99	0.00	0.015	0.02	0.33
Briskness	Moan	-0.07	0.11	-0.59	2785.01	0.56	1.00	-0.37	0.24
Briskness	Scream	0.39	0.11	3.65	2794.47	<.001	0.002	0.10	0.68
Briskness	Sigh	0.06	0.06	1.03	2807.45	0.30	1.00	-0.10	0.23
Novelty	Gasp	0.08	0.11	0.75	2773.64	0.45	1.00	-0.21	0.37
Novelty	Groan	-0.09	0.10	-0.93	2777.04	0.35	1.00	-0.37	0.18
Novelty	Grunt	-0.06	0.12	-0.48	2779.49	0.63	1.00	-0.37	0.26
Novelty	Laugh	0.02	0.06	0.28	2834.11	0.78	1.00	-0.15	0.19
Novelty	Moan	0.12	0.14	0.81	2784.68	0.42	1.00	-0.27	0.50
Novelty	Scream	0.24	0.10	2.44	2777.57	0.02	0.10	-0.03	0.51
Novelty	Sigh	-0.10	0.07	-1.41	2805.28	0.16	0.95	-0.28	0.09

Note. β = unstandardised slope (change in appropriateness units per +1 SD in the dimension); SE = standard error; CI = 95% confidence interval; *p* values are Holm-Bonferroni adjusted within each dimension ($n = 7$). Slopes and CIs were obtained via `emtrends` in the *emmeans* package with Satterthwaite df. Significant cells are those with adjusted $p < .05$

Table S10 Summary of hypothesis tests

Hyp.	Predictor	Test statistic	Effect size	p	Key finding
**H1**	Vocalisation type	$F(9, 3721) = 101.19$	$f = 0.495$	<.001	Laugh most appropriate ($M = 7.55$); retch least ($M = 4.74$). Accounted for 16.1% of variance.
**H2a**	Location	$F(3, 3694) = 38.83$	$f = 0.178$	<.001	Home and social places rated higher than work/school and public places ($d = 0.35$–0.44). 2.7% of variance.
**H2b**	Interaction partner	$F(3, 4386) = 25.98$	$f = 0.133$	<.001	Close ties rated highest; strangers and weak ties lowest ($d = 0.31$–0.36). 1.7% of variance.
**H2c**	Psychological dimensions	$\chi^2(14) = 233.88$	$R^2m = .072$	<.001	Formality and power associated with lower appropriateness; sociality and positivity with higher. 7.2% of variance.
**H3a**	Vocalisation × Location	$F(18, 3327) = 5.66$	$\eta^2 = .030$	<.001	Laugh showed strongest location sensitivity (highest in social places). Sigh highest at home. Grunt showed minimal variation.
**H3b**	Vocalisation × Partner	$F(27, 4259) = 5.92$	$\eta^2 = .036$	<.001	Laugh highest with close ties. Sigh highest when alone. Scream higher with close ties than strangers.
**H3c**	Vocalisation × Psych. dim.	See Table S9	See Table S9		Laugh more appropriate in positive, social, brisk contexts; less in formal and high-power. Scream more appropriate in positive, social, brisk contexts. Grunt less appropriate as sociality increased.

Note. f = Cohen's f; η^2 = eta-squared for interaction term; R^2_m = marginal R-squared (variance explained by fixed effects). All models included random intercepts for participants. Detailed results for each hypothesis are reported in the main text and Supplementary Tables S7–S9.

Table S11. Effect of nationality (Dutch vs. non-Dutch) as a between-person covariate across all hypothesis models

Model	β	SE	*t*	*p*
H1 (Vocalisation type)	0.349	0.185	1.89	.061
H2a (Location)	0.430	0.194	2.22	.028
H2b (Interaction partner)	0.400	0.196	2.04	.043
H2c (Psychological dimensions)	0.362	0.189	1.91	.058
H3a (Vocalisation × Location)	0.321	0.197	1.63	.106
H3b (Vocalisation × Partner)	0.288	0.195	1.47	.143
H3c (Vocalisation × Psych. dimensions)	0.246	0.184	1.33	.184

Note. Nationality was coded as 1 = Dutch (n = 42), 0 = non-Dutch (n = 100). Positive estimates indicate that Dutch participants rated appropriateness higher. All substantive fixed effects from the original models remained unchanged in direction, magnitude, and significance when nationality was included.

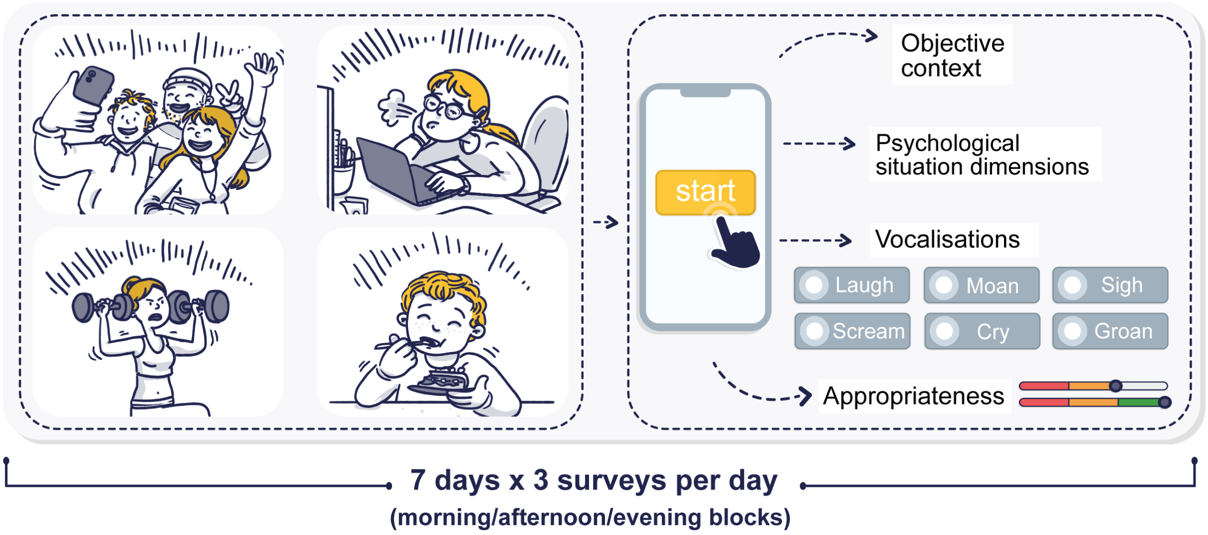

Figure S1. Experience-sampling procedure. Participants received three daily smartphone prompts for seven consecutive days, scheduled randomly within morning (09:00–13:00), afternoon (13:00–18:00), and evening (18:00–23:00) blocks. Each survey assessed situational features, observed vocalisations, and perceived appropriateness.

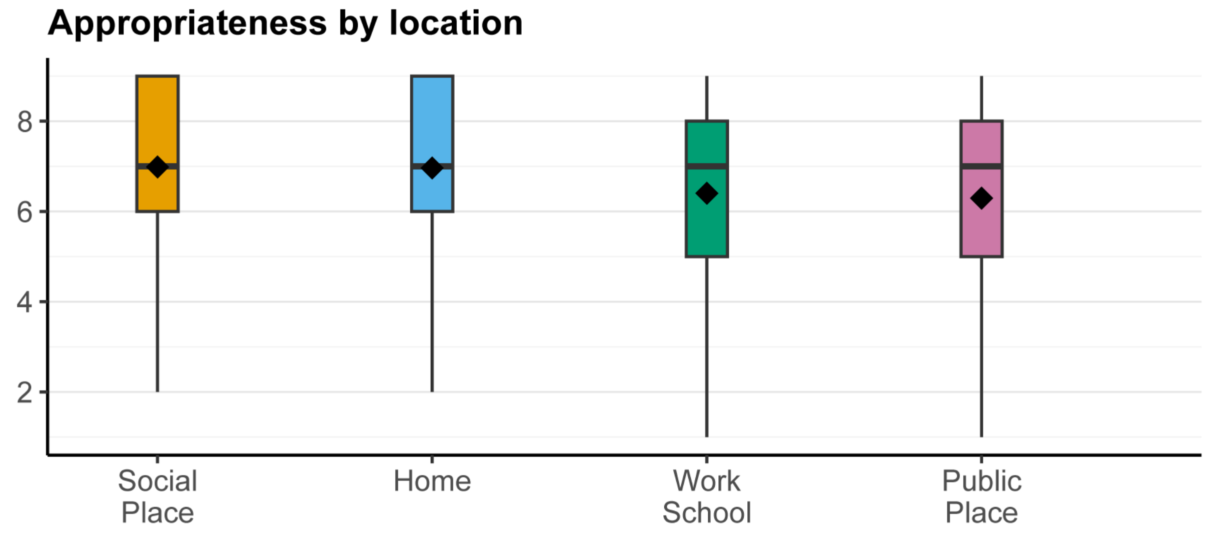

Figure S2. Boxplots showing appropriateness by location, with boxes representing interquartile ranges, horizontal lines showing medians, and diamonds indicating means; social places and home rated higher than work/school and public places.

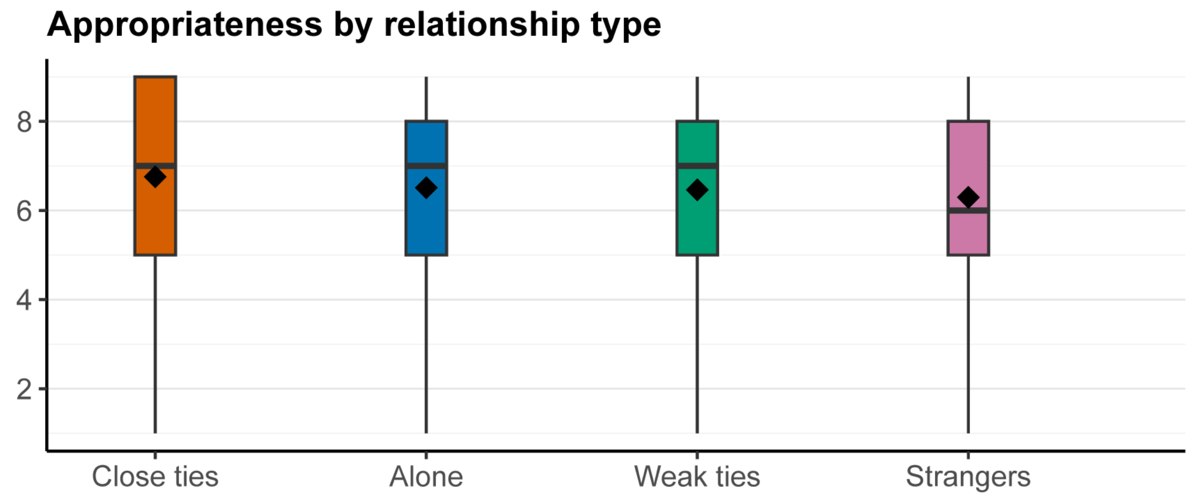

Figure S3. Boxplots showing appropriateness by interaction partner, with close ties highest and strangers lowest. Boxes represent interquartile ranges, horizontal lines showing medians, and diamonds indicating means.

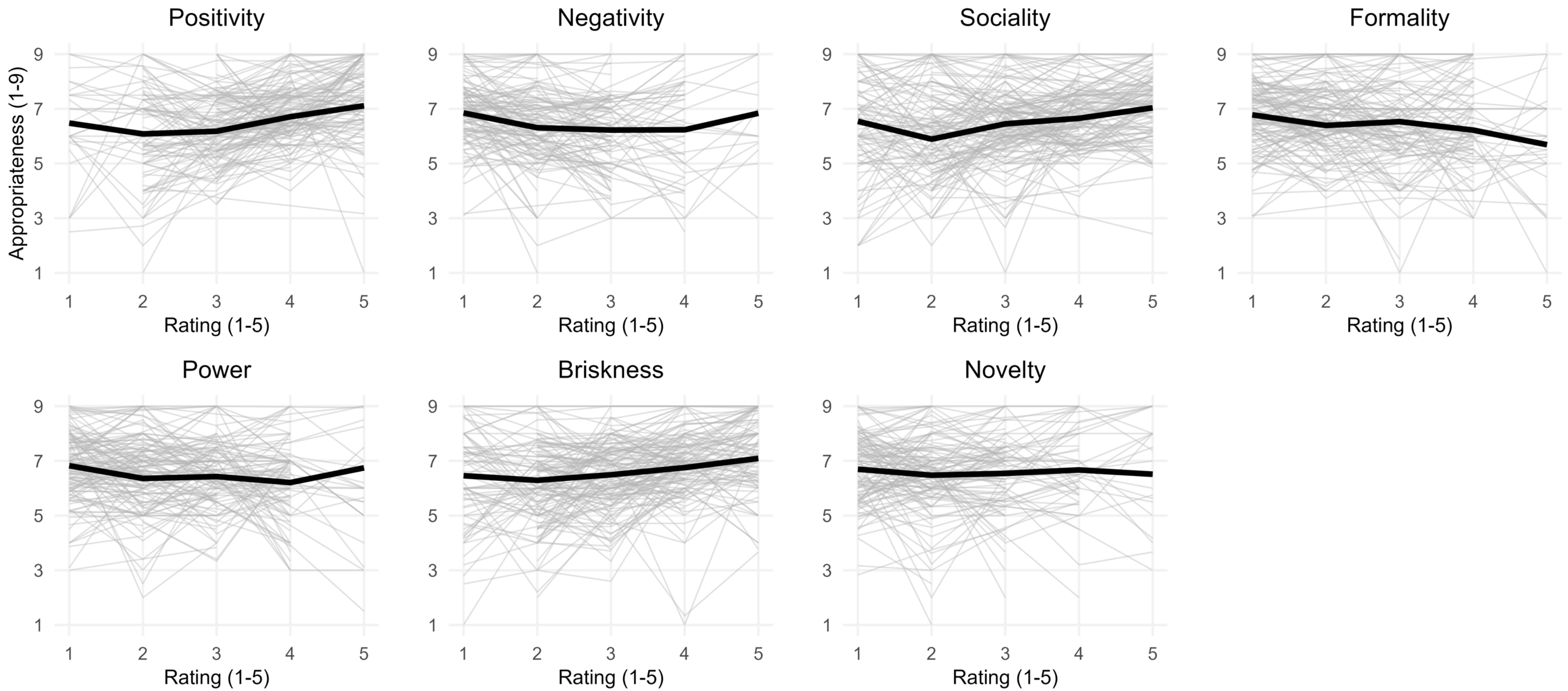

Figure S4. Within-person trajectories of psychological situation dimensions across the experience sampling period. Each grey line represents an individual participant's ratings (1-5 scale) across surveys, with thick black lines showing overall trends. Plots demonstrate substantial within-person variability across all seven dimensions, supporting the multilevel modelling approach. Visible trends align with main effects results: Formality and Power show downward slopes (negative coefficients), while Sociality and Positivity show slight upward trends (positive coefficients). Negativity, Briskness, and Novelty show relatively flat trajectories, consistent with their non-significant effects in predicting appropriateness ratings.

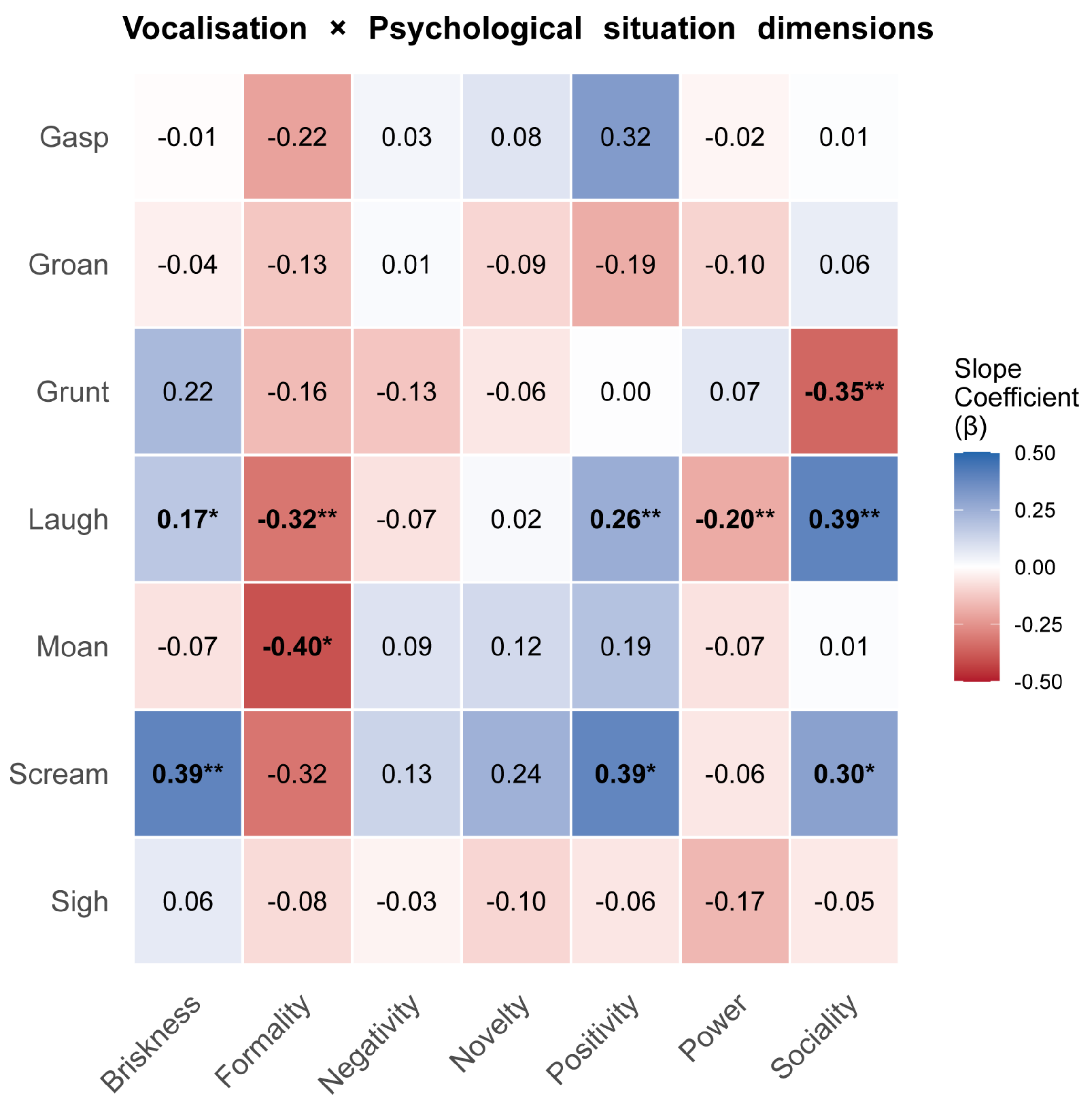

Figure S5. Heatmap of simple-slope coefficients (β) from linear mixed-effects models for each psychological characteristic × vocalisation. Cells display standardised β (diverging scale centred at 0); Asterisks mark Holm-corrected significance (* $p < .05$, ** $p < .01$). Full statistics (β, SE, t, df, raw p, Holm-adjusted p, 95% CI) are provided in Table S9.
